# The association between sleep parameters, cognitive functioning, and markers of brain morphology: *The Maastricht Study*

**DOI:** 10.1093/sleep/zsaf218

**Published:** 2025-07-28

**Authors:** Tessa L van Baal, Sebastian Köhler, Annemarie Koster, Ree M Meertens, Hans Bosma, Miranda T Schram, Jacobus F A Jansen, Sami O Simons, Bastiaan E de Galan, Simone J P M Eussen, Niels Janssen, Kay Deckers

**Affiliations:** Mental Health and Neuroscience Research Institute (MHeNs), Maastricht University, Maastricht, The Netherlands; Alzheimer Center Limburg, Department of Psychiatry and Neuropsychology, Maastricht University, Maastricht, The Netherlands; Mental Health and Neuroscience Research Institute (MHeNs), Maastricht University, Maastricht, The Netherlands; Alzheimer Center Limburg, Department of Psychiatry and Neuropsychology, Maastricht University, Maastricht, The Netherlands; Care and Public Health Research Institute (CAPHRI), Maastricht University, Maastricht, The Netherlands; Department of Social Medicine, Maastricht University, Maastricht, The Netherlands; Care and Public Health Research Institute (CAPHRI), Maastricht University, Maastricht, The Netherlands; Institute of Nutrition and Translational Research in Metabolism (NUTRIM), Maastricht University, Maastricht, The Netherlands; Department of Health Promotion, Maastricht University, Maastricht, The Netherlands; Care and Public Health Research Institute (CAPHRI), Maastricht University, Maastricht, The Netherlands; Department of Social Medicine, Maastricht University, Maastricht, The Netherlands; Mental Health and Neuroscience Research Institute (MHeNs), Maastricht University, Maastricht, The Netherlands; Department of Internal Medicine, Maastricht University Medical Center+, Maastricht, The Netherlands; Cardiovascular Research Institute Maastricht (CARIM), Maastricht University, Maastricht, The Netherlands; Heart and Vascular Centre, Maastricht University Medical Center+, Maastricht, The Netherlands; Department of Epidemiology, Erasmus Medical Center, Rotterdam, The Netherlands; Mental Health and Neuroscience Research Institute (MHeNs), Maastricht University, Maastricht, The Netherlands; Department of Radiology and Nuclear Medicine, Maastricht University Medical Center+, Maastricht, The Netherlands; Department of Electrical Engineering, Eindhoven University of Technology, Eindhoven, The Netherlands; Institute of Nutrition and Translational Research in Metabolism (NUTRIM), Maastricht University, Maastricht, The Netherlands; Department of Respiratory Medicine, Maastricht University Medical Center+, Maastricht, The Netherlands; Department of Internal Medicine, Maastricht University Medical Center+, Maastricht, The Netherlands; Cardiovascular Research Institute Maastricht (CARIM), Maastricht University, Maastricht, The Netherlands; Department of Internal Medicine, Radboud University Medical Center, Nijmegen, The Netherlands; Care and Public Health Research Institute (CAPHRI), Maastricht University, Maastricht, The Netherlands; Cardiovascular Research Institute Maastricht (CARIM), Maastricht University, Maastricht, The Netherlands; Department of Epidemiology, Maastricht University, Maastricht, The Netherlands; Mental Health and Neuroscience Research Institute (MHeNs), Maastricht University, Maastricht, The Netherlands; Alzheimer Center Limburg, Department of Psychiatry and Neuropsychology, Maastricht University, Maastricht, The Netherlands; Mental Health and Neuroscience Research Institute (MHeNs), Maastricht University, Maastricht, The Netherlands; Alzheimer Center Limburg, Department of Psychiatry and Neuropsychology, Maastricht University, Maastricht, The Netherlands

**Keywords:** cognitive function, accelerometry, neuroimaging, dementia risk reduction, epidemiology

## Abstract

**Study Objectives:**

To examine the association of objective and subjective sleep parameters with cognitive functioning and markers of brain morphology.

**Methods:**

This cross-sectional study included 3360 participants (mean age: 59.5 ± 8.5 years; 51.1% female) from The Maastricht Study. Time in bed (TIB) and sleep breaks were objectively estimated using a thigh-worn accelerometer. Subjective sleep continuity was assessed via a single-item, and excessive daytime sleepiness was evaluated using the Epworth Sleepiness Scale. Cognitive testing was administered across three domains—memory, information processing speed, and executive functioning and attention. Markers of brain morphology (e.g. hippocampal volume, gray matter (GM) volume, and white matter volume) were assessed with 3 Tesla magnetic resonance imaging. Linear and logistic regression analyses modeled the associations.

**Results:**

A longer TIB (h/night) was associated with worse executive functioning and attention (B_linear_ = −0.052, 95% CI = −0.084 to −0.019). Categorical analyses showed that a longer TIB (≥9 h/night) was associated with worse executive functioning and attention (B = −0.088, 95% CI = −0.166 to −0.010) compared to a mid-range TIB (≥7 to <9 h/night). A curvilinear association was found between TIB and lower GM volume (B_quadratic_ = −0.013, 95% CI = −0.025 to −0.001). Sleep breaks (≥2/night) were associated with worse overall cognition (B = −0.069, 95% CI = −0.124 to −0.013), information processing speed (B = −0.125, 95% CI = −0.212 to −0.039), and reduced GM volume (B = −0.068, 95% CI = −0.118 to −0.018). No significant associations were found for memory or other markers of brain morphology. Subjective sleep parameters showed no associations with cognitive functioning or markers of brain morphology.

**Conclusions:**

Adequate and uninterrupted TIB was associated with better cognitive functioning and brain morphology.

**Statement of Significance**

With the growing prevalence of dementia, the importance of reducing dementia risk is increasingly acknowledged. Sleep has been identified as a potential risk factor for cognitive decline and dementia, yet studies using objective measures of sleep remain limited. This study explores subjective and objective sleep parameters in relation to cognitive function and brain morphology in a large cohort. The findings show that a long time in bed (TIB) is negatively associated with executive functioning and attention, alongside a curvilinear relationship between TIB and gray matter volume. Additionally, sleep breaks (≥2/night) are linked to lower brain volume and poorer cognition. Future research should investigate longitudinal associations and other sleep aspects, including regularity and sleep disorders, to further our understanding and guide dementia prevention strategies.

## Graphical Abstract

**Graphical Abstract:**
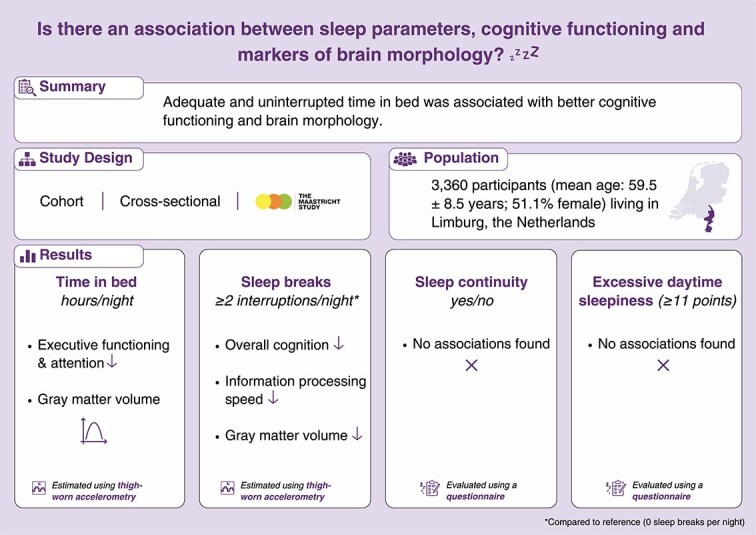

In 2019, it was estimated that 57.4 million individuals worldwide lived with dementia [1]. Given the prevailing trends in population aging and overall population growth, this number was projected to increase to an estimated 152.8 million cases by 2050. This trend profoundly impacts individuals with dementia, as well as their caregivers and social networks, while also placing a substantial burden on economies, societies, and healthcare infrastructures [2]. Prioritizing dementia risk reduction at both population and individual levels is thus a promising strategy to tackle these burdens [3], alongside ongoing research efforts to halt the underlying disease progression pharmacologically.

Observational research has highlighted that lifestyle factors such as abstaining from smoking and engaging in regular physical activity may play a pivotal role in reducing the risk of dementia [3–5], regardless of genetic susceptibility [6]. Sleep has also emerged as a promising modifiable risk factor, with an umbrella review and Delphi study recommending its inclusion in dementia risk assessments [7]. As a multidimensional construct, sleep encompasses various aspects, such as duration, quality, and timing [8], prompting research to explore a wide range of sleep parameters and their associations with cognitive health and dementia. For example, a meta-analysis of 51 longitudinal cohort studies revealed that various self-reported sleep parameters, such as excessive daytime sleepiness, sleep fragmentation, and short as well as long sleep durations, were associated with an increased risk of all-cause cognitive disorders or dementia [9]. In addition to these commonly investigated parameters, time in bed (TIB) serves as a complementary yet distinct measure to sleep duration [10], representing an individual’s sleep opportunity [11] rather than the total time asleep. Notably, a longitudinal study using data from the Shandong Yanggu Study of Aging and Dementia and the Multimodal Interventions to Delay Dementia and Disability in Rural China (MIND-China) found that, among older adults in rural China, prolonged TIB is associated with an elevated risk of dementia and accelerated cognitive decline [12].

Moreover, there is growing interest in the potential influence of sleep on brain morphology [13], with reported associations involving hippocampal volume [14], gray matter (GM) [15], white matter (WM) microstructure [16], and cerebral small vessel disease (CSVD) [17]. For example, a prospective cohort study from the Coronary Artery Risk Development in Young Adults study, which included 613 participants, reported that individuals with a short sleep duration (≤6 h), compared to those with a mid-range sleep duration (>6 to ≤8 h), had a significantly higher ratio of white matter hyperintensities (WMH) to normal tissue in the parietal region [18]. Additionally, accelerometer-derived sleep fragmentation has been associated with lower GM volume in lateral orbital and inferior frontal regions [19].

Despite the growing evidence supporting the link between sleep and dementia [20–24], the absence of sleep as a listed modifiable risk factor in the 2024 report from the Lancet Commission on Dementia Prevention, Intervention, and Care [3] highlights the ongoing ambiguity surrounding the relationship between sleep, cognitive decline, and dementia. Most studies to date have relied on self-reported sleep measures, with fewer incorporating objective measures, and even fewer employing both in tandem. While subjective measures capture individual sleep perceptions, they are prone to information bias. Objective measures, by contrast, provide quantifiable parameters of sleep. As a result, there is a growing consensus that combining both approaches offers a more comprehensive and nuanced characterization of sleep [25–27]. In studies using objective sleep parameters, an additional gap lies in the limited examination of nonlinear associations with objective total sleep time or TIB and cognitive outcomes [28]. Finally, there is a notable lack of research incorporating sleep measures, comprehensive neuropsychological assessments, and neuroimaging data to explore the associations among sleep, cognition, and brain morphology.

This study, therefore, aims to investigate the cross-sectional associations between objective (TIB and sleep breaks) and subjective (sleep continuity and excessive daytime sleepiness) sleep parameters with cognitive functioning as well as various markers of brain morphology (e.g. GM volume and CSVD) within an adult population. It is hypothesized that shorter and longer TIB, increased sleep breaks and excessive daytime sleepiness are associated with worse cognitive function and brain morphology.

## Materials and Methods

### Study population

Data were obtained from The Maastricht Study, an ongoing prospective population-based cohort study. The rationale and methodology of the study have been previously described [29]. In short, the study focuses on the etiology, pathophysiology, complications, and comorbidities of type 2 diabetes mellitus (T2DM) and is characterized by an extensive phenotyping approach. Individuals aged between 40 and 75 years living in the southern region of the Netherlands (province of Limburg) were eligible to participate. Participants were recruited through mass media campaigns, municipal registries, and the regional Diabetes Patient Registry through mailings. Recruitment was stratified according to known T2DM status, with an oversampling of individuals with T2DM for efficiency reasons. The present study includes cross-sectional data from 9187 participants who completed the baseline survey between November 2010 and October 2020. The examinations of each participant were performed within a three-month timeframe, and magnetic resonance imaging (MRI) measurements of the brain were incorporated from December 2013 onwards. The study included individuals who met the following additional criteria: (1) availability of valid accelerometry/TIB data, (2) availability of valid MRI data, (3) presence of data on at least 11 modifiable risk and protective factors from the original LIfestyle for BRAin Health (LIBRA1) [7], (4) availability of socioeconomic position (SEP) data, yielding a final study population of 3360. The study was approved by the institutional medical ethical committee (NL31329.068.10) and the Ministry of Health, Welfare and Sports of the Netherlands (Permit 131 088-105 234-PG). All participants gave written informed consent. The current study is reported in accordance with the standards of the STrengthening Reporting of OBservational studies in Epidemiology (STROBE) guidelines (Supplementary Table S1).

### Measurements

#### Sleep

##### Accelerometry data

TIB was operationalized as the mean total nighttime minutes in bed, with the mean duration of active periods (stepping and standing) at night subtracted from this total. The algorithm to determine TIB and sleeping breaks has been previously detailed [30]. Bedtime was based on the frequency and duration of sedentary bouts of sitting/lying time within a window from 19:00 to 12:00 (noon), in line with self-reported data [30]. However, within this study, the average minutes of stepping and standing during the night were also subtracted from the calculated TIB. Participants were categorized into groups based on their TIB in accordance with the recommendations of sleep duration by The National Sleep Foundation [31]. The reference group, defined as having a mid-range TIB, was characterized as having between ≥7 and <9 h of TIB per night. A short TIB was categorized as <7 h per night, while a long TIB referred to individuals with ≥9 h of TIB per night.

Sleep breaks were operationalized using data on the mean number of sleep breaks at night. Sleep breaks were defined as interruptions from a sitting or lying position lasting at least 1 minute [30]. Based on the average frequency of sleep breaks at night, three numerical categories were established: 0, 1, and ≥2 sleep breaks at night.

These parameters were measured using the activPAL3™ physical activity monitor (PAL Technologies, Glasgow, UK) [32]. The activPAL3™ is a small (53 × 35 × 7 mm), lightweight (15 g) triaxial accelerometer that records a participant’s movement in the vertical, anteroposterior, and mediolateral axes, as well as determining posture (sitting, lying, standing, and stepping) based on acceleration information. The activPAL3™ was directly attached to the skin of the anterior right thigh using a transparent 3M Tegaderm tape, following waterproofing with a nitrile sleeve. Participants were asked to wear accelerometers for eight consecutive days without removing the device at any time. To prevent the misidentification of non-wear time, participants were asked not to replace the device once removed. Data were uploaded using the activPAL3™ software and processed using customized software written in MATLAB R2018b (MathWorks, Natick, MA, USA) [30]. Data from the first wear day were excluded as participants were asked to perform physical activity tests. Additionally, data from the final wear day were included in the analysis if more than 14 h of data were recorded.

##### Self-reported measurements

Sleep continuity (yes/no) was evaluated through the single-item question, “Do you sleep continuously?” Excessive daytime sleepiness was assessed using the Epworth Sleepiness Scale (ESS) [33], a self-reported questionnaire that measures a subject’s habitual likelihood of dozing or falling asleep in common daily living situations. The ESS is an 8-item scale (each item scored from 0 to 3) with a maximum score of 24. Individuals with values ≥11 were considered to have excessive daytime sleepiness [33]. The ESS has demonstrated strong reliability and validity [34–36].

### Cognitive functioning

Trained research assistants administered a 30-min battery of neuropsychological tests to assess cognitive functioning in three domains: memory, information processing speed, and executive functioning and attention [29]. The memory function domain score was calculated by averaging immediate and delayed recall scores from the Verbal Learning Test [37]. A domain score for information processing speed was determined using standardized scores from the Stroop Color-Word Test Part I and II [38], Concept Shifting Test Parts A and B [39], and Letter-Digit Substitution Test [40]. The executive function and attention domain score was calculated from the average scores of the Stroop Color-Word Test Part III and Concept Shifting Test Part C. Raw individual test scores were standardized into *z*-scores based on the mean and standard deviation (SD) from the whole study sample. These *z*-scores were then averaged to create cognitive domain scores, with higher scores indicating better performance. Subsequently, the three domain scores were further averaged to derive an overall cognition score.

### Markers of brain morphology

Markers of brain morphology were evaluated using volumetric parameters. Brain MRI was conducted utilizing a 3-Tesla clinical MRI scanner (MAGNETOM Prisma^fit^ Syngo MR D13D; Siemens Healthcare, Erlangen, Germany) using a head/neck coil equipped with 64 elements for parallel imaging. An ISO-13485:2012 certified automated method, including visual inspection, was employed to analyze T1 images and T2-weighted fluid-attenuated inversion recovery (FLAIR) images [41, 42]. T1 images were segmented to obtain cerebrospinal fluid (CSF), GM, and WM volumes (1 voxel = 1.00 mm^3^ = 0.001 mL) [42], with CSF serving as an inverted measure of brain atrophy. Total hippocampal volume was yielded from T1 images through automated segmentation of hippocampal subfields [43] using FreeSurfer v6 [44]. Intracranial volume (ICV) was calculated as the sum of CSF, GM, and WM. T1 images and T2-weighted FLAIR images were used to calculate WMH volume [41]. WMHs were also visually rated using the Fazekas scale [45]. WMH volume was log-transformed to reduce skewness, and all volumetric measurements were standardized. Three neuroradiologists manually counted cerebral lacunar infarcts and cerebral microbleeds following the Microbleed Anatomical Rating Scale [46, 47]. The presence of CSVD (yes/no) was established based on one of the following criteria: (1) a Fazekas score ≥ 2; (2) the presence of cerebral lacunar infarcts; or (3) the presence of cerebral microbleeds [48].

### Demographics and covariates

Information on age (years), sex (male/female), and SEP (low, middle, high) was collected from questionnaires. SEP was evaluated using a composite score comprising educational attainment, occupational status, and household income [49]. Educational attainment was determined by the participants’ highest level of completed education. These levels were initially categorized into nine groups and then consolidated into three categories: low (levels 1–4: no education, incomplete or completed primary education, or lower vocational education), middle (levels 5–7: intermediate vocational education or higher secondary education), and high (levels 8–9: higher vocational education or university education). Occupational status was measured using the International Standard Classification of Occupations 2008 (ISCO-08), a hierarchical framework that organizes occupations based on the education and skills required for a given role [50–52]. The resulting scores were standardized into *z*-scores and divided into tertiles. The classifications of the International Socioeconomic Index (ISEI-08) were categorized into low, middle, and high based on these tertiles. Household income was assessed using self-reported monthly net income, provided in 19 categories ranging from 0 to >5000 euros per month, along with household size [53]. To account for household size, the net income was divided by the square root of the number of household members and then standardized into a measure of household income equivalent. The standardized income was divided into tertiles to classify household income into low, middle, and high groups. A composite SEP score was calculated by standardizing the scores for educational attainment, occupational status, and household income. The standardized scores were then averaged and divided into tertiles representing low, middle, and high SEP. The use of sleep (affecting) medication (yes/no) was evaluated during a medication interview where the generic name, dosage, and frequency were registered [29]. Clinical factors and lifestyle behaviors associated with cognitive decline and dementia risk were summarized using the original LIBRA1 index [5]. LIBRA1 consists of 12 individual risk and protective factors for dementia. Risk factors include physical inactivity, smoking, obesity, depression, T2DM, hypertension, hypercholesterolaemia, heart disease, and chronic kidney disease. Meanwhile, protective factors include high engagement in cognitive activity, adherence to a Mediterranean diet, and low to moderate alcohol intake. All LIBRA1 factors could be operationalized in The Maastricht Study (based on both objective data and validated questionnaires) except for high engagement in cognitive activity, which was excluded from the scoring due to the unavailability of data. The LIBRA1 total score is determined by assigning a weight to each factor, with a positive weight indicating the presence of risk factors and a negative weight indicating the presence of protective factors. In the current study, the adjusted LIBRA1 score, encompassing 11 factors, ranges from −2.7 to +12.7, with higher scores indicating a worse dementia risk profile. See Supplementary Table S2 for further detailed information regarding the operationalization of LIBRA1 factors.

### Statistical analyses

The study population’s descriptive characteristics were summarized as mean ± SD or frequencies and percentages. Differences in characteristics between the three TIB groups, as well as differences between the final study sample and the excluded group, were tested using one-way analysis of variance or *t*-tests for continuous variables and Pearson’s *χ*^2^ test for categorical variables.

Separate multiple linear regression models were used to test for an association between TIB (continuous and categorical), sleep breaks, sleep continuity, excessive daytime sleepiness, and outcomes: (1) overall and domain-specific cognitive functioning and (2) markers of brain morphology (GM volume, WM volume, hippocampal volume, CSF and WMH volume). Binary logistic regression analyses were used to investigate the association between objective and subjective sleep parameters and the presence of CSVD. All analyses followed the same protocol of consecutively adding blocks of covariates in a stepwise manner. In Model 1, demographic factors (age, sex, SEP) and T2DM were adjusted for. Model 2 was additionally adjusted for LIBRA1 scores, with T2DM removed from the model as it is included within LIBRA1. In the main model (Model 3), sleep medication was included as an additional covariate. When sleep breaks were considered an independent variable, the main model also included TIB. When volumetric markers of brain morphology and the presence of CSVD were outcomes, the crude model also adjusted for MRI lag time (correcting for the time between assessment and the MRI) and ICV (correcting for head size, volumetric markers only).

Since the association between TIB and cognitive functioning may be nonlinear, as previously described in the literature using sleep duration [54], a quadratic term of TIB was entered into Model 3 to test for nonlinear associations. A likelihood ratio (LR) test was applied to compare the fit of the linear versus the quadratic (i.e. curvilinear) Model 3. Non-significant *p*-values suggest no deviation from a linear trend.

Associations are expressed by unstandardized regression coefficients (B) and 95% confidence intervals (CI) for linear regressions, odds ratios (OR), and 95% CI for logistic regressions.

#### Additional analyses

Separate interaction analyses were performed to examine the potential moderating effects of age, sex, composite SEP score, and T2DM on significant associations in the main model. Interaction analyses for T2DM were performed due to the oversampling of participants with T2DM. In case of significance (*p* < .05), stratified analyses were also conducted to provide more detailed insight into the nature of differences between strata. If age exhibits a significant interaction, stratified analyses will be conducted in two groups: ≤60 years and >60 years. Research indicates that sleep parameters decline until the age of 60, after which they remain relatively stable [55].

#### Sensitivity analyses

A sensitivity analysis was conducted using Model 3 for the cognitive functioning analyses, including all participants regardless of MRI data availability, to assess the potential impact of selection bias and the risk of false negatives. Moreover, a sensitivity analysis was performed using Model 3, accounting for the components (educational attainment, occupational status, and household income) of the composite SEP score separately. This approach may enhance the robustness of the findings and help identify which SEP component is driving potentially observed associations. All data were analyzed using Stata statistical software, version 17.0 (StataCorp., College Station, Texas, United States of America). A two-tailed *p*-value of *<*.05 was considered statistically significant.

## Results

### General characteristics of the study population

**Figure 1:**
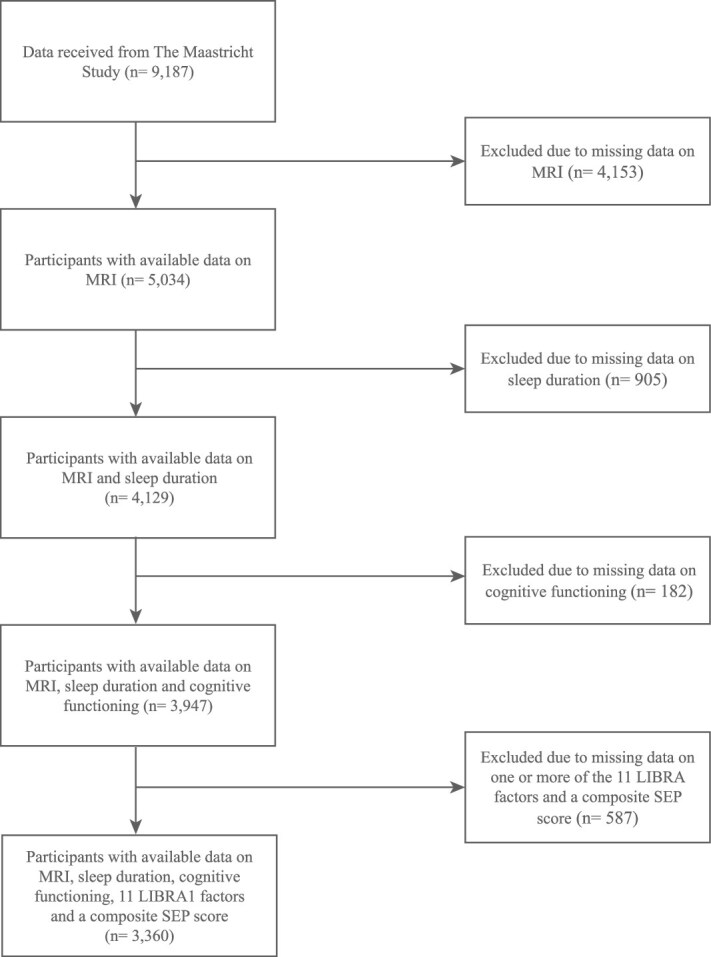
Flowchart of participant inclusion and exclusion. Abbreviations: MRI, magnetic resonance imaging; LIBRA1, original LIfestyle for BRAin health score; SEP, socioeconomic position.

Out of the 9187 participants, 5827 (63.4%) were excluded from this study due to missing data on one or more variables (Figure 1), most of which (45.2% of the total sample) were due to unavailable MRI scans or participants having MRI contraindications. The final analytical sample included 3360 participants (mean age: 59.5 ± 8.5 years; 51.1% female).

Excluded participants (*n* = 5827) had lower composite SEP scores (35.1% vs 30.3%; *χ*^2^ [2] = 24.2, *p* < .001), a greater prevalence of T2DM (23.4% vs 19.1%; *χ*^2^ [1] = 22.9, *p* < .001) and an unhealthier lifestyle indicated by a higher mean LIBRA1 score (1.6 vs 1.2; *t*[6417] = 7.8, *p* < .001) compared to the included sample. Additionally, excluded participants had more sleep breaks (≥2/night) (16.0% vs 11.8%; *χ*^2^[2] = 47.7, *p <* .001), reported less sleep continuity (59.6% vs 62.6%; *χ*^2^ [1] = 7.4, *p* = .007), scored lower on cognition scores: overall cognition (−0.03 vs 0.09; *t*[8620] = −7.8, *p* < .001), memory (−0.06 vs 0.10; *t*[8783] = −7.3, *p* < .001), information processing speed (−0.06 vs 0.10; *t*[8722] = −7.6, *p* < .001), and executive functioning and attention (−0.05 vs 0.08; *t*[8687] = −6.0, *p* < .001), and demonstrated a higher volume of WMH (1.2 mL vs 0.9 mL; *t*[5181] = 3.0, *p* = .002). See Supplementary Table S3 for a more detailed overview of the differences in characteristics.

Table 1 shows the characteristics of the total study population, stratified by TIB. Individuals with a long TIB were more often female (57.6%) and had a higher proportion of low SEP (37.0%), while those with a short TIB more frequently had a high SEP (39.9%) compared to a long SEP (*p* < .001). LIBRA1 scores were slightly higher in both short (1.4) and long (1.5) TIB groups compared to the mid-range group (1.1), indicating a greater (modifiable) dementia risk. A long TIB was also associated with more sleep breaks compared to mid-range and short TIB, lower overall cognitive performance (*z*-score: 0.01 vs short: 0.11; mid-range: 0.10), and smaller brain volumes, for example, GM volume was lower in the long TIB group (650.5 mL) compared to the short (668.3 mL) and mid-range (662.4 mL) groups.

**Table 1:** Demographic and Health Characteristics of the Study Population Stratified by Categories of TIB

		Time in bed (categories)			
Characteristics	Total *n* = 3360	Short (<7 h/night) *n* = 266	Mid-range (≥7 to <9 h/night) *n* = 2545	Long (≥9 h/night) *n* = 549	*p*
***Demographics***					
Age, mean (SD)	59.5 (8.5)	59.0 (8.1)	59.4 (8.5)	60.4 (8.5)	.028
Female, *n* (%)	1716 (51.1)	112 (42.1)	1288 (50.6)	316 (57.6)	<.001
Composite SEP score^*^, *n* (%)					<.001
Low	1018 (30.3)	76 (28.6)	739 (29.0)	203 (37.0)	
Middle	1199 (35.7)	84 (31.6)	916 (34.0)	199 (36.3)	
High	1143 (34.0)	106 (39.9)	890 (35.0)	147 (26.8)	
LIBRA1 score, mean (SD)	1.2 (2.1)	1.4 (2.1)	1.1 (2.1)	1.5 (2.1)	<.001
Individual LIBRA1 factors, *n* (%)					
Obesity	589 (17.5)	56 (21.1)	426 (16.7)	107 (19.5)	.089
Physical inactivity	865 (25.7)	58 (21.8)	633 (24.9)	174 (31.7)	.001
Adherence to a Mediterranean diet	965 (28.7)	75 (28.2)	734 (28.8)	156 (28.4)	.961
Smoking	372 (11.1)	38 (14.3)	266 (10.5)	68 (12.4)	.093
Low to moderate alcohol intake	1838 (54.7)	149 (56.0)	1378 (54.2)	238 (43.4)	.511
T2DM	642 (19.1)	69 (25.9)	456 (17.9)	117 (21.3)	.002
Hypertension	1701 (50.6)	147 (55.3)	1265 (49.7)	289 (52.6)	.132
Depression	94 (2.8)	8 (3.0)	61 (2.4)	25 (4.6)	.021
Heart disease	180 (5.4)	17 (6.4)	135 (5.3)	28 (5.1)	.724
Hypercholesteremia	517 (15.4)	24 (9.0)	391 (15.4)	102 (18.6)	.002
Chronic kidney disease	396 (11.8)	35 (13.2)	280 (11.0)	81 (14.8)	.036
Sleep medication, *n* (%)	72 (2.1)	5 (1.9)	54 (2.1)	13 (2.4)	.893
***Objective sleep measures***					
Time in bed (h/night),^†^ mean (SD)	8.2 (0.9)	6.5 (0.4)	8.1 (0.5)	9.5 (0.5)	<.001
Sleep breaks (per night),^‡^ *n* (%)					<.001
0	1698 (50.5)	123 (46.2)	1364 (53.6)	211 (38.4)	
1	1267 (37.7)	103 (38.7)	908 (35.7)	256 (46.6)	
≥2	395 (11.8)	40 (15.0)	273 (10.7)	82 (14.9)	
***Subjective sleep measures***					
Sleep continuity (yes), *n* (%)^§^	2085 (62.6)	177 (66.8)	1600 (63.4)	308 (56.6)	.004
Excessive daytime sleepiness (score ≥ 11), *n* (%)^||^	461 (13.8)	55 (20.8)	343 (13.7)	61 (11.2)	.001
***Cognitive functioning***					
Overall cognition, mean (SD)	0.09 (0.63)	0.11 (0.69)	0.10 (0.62)	0.01 (0.65)	.011
Memory, mean (SD)	0.10 (0.95)	0.11 (0.97)	0.10 (0.95)	0.08 (0.97)	.901
Information processing speed, mean (SD)	0.10 (0.93)	0.07 (1.05)	0.13 (0.90)	0.00 (0.96)	.012
Executive functioning and attention, mean (SD)	0.08 (0.95)	0.18 (1.12)	0.10 (0.92)	−0.07 (0.99)	<.001
***Markers of brain morphology***					
Gray matter volume (mL), mean (SD)	660.9 (59.4)	668.3 (59.1)	662.4 (59.4)	650.5 (58.9)	<.001
White matter volume (mL), mean (SD)	476.0 (58.1)	482.6 (58.7)	476.8 (58.2)	469.1 (57.2)	.003
Right hippocampal volume (mm^3^), mean (SD)	3428.5 (353.4)	3455.1 (345.4)	3436.4 (356.1)	3378.6 (340.2)	.001
Left hippocampal volume, mean (mm^3^)	3410.6 (351.8)	3438.1 (347.4)	3415.4 (354.9)	3368.8 (336.9)	.007
Total hippocampal volume, mean (mm^3^)	6838.1 (689.0)	6893.2 (675.2)	6851.8 (694.5)	6747.5 (663.0)	.002
White matter hyperintensities volume (mL), mean (SD)	0.4 (0.5)	0.4 (0.5)	0.4 (0.5)	0.4 (0.5)	.871
Cerebrospinal fluid volume (mL), mean (SD)	251.8 (46.8)	253.7 (46.5)	251.7 (46.9)	251.2 (46.7)	.768
Presence of cerebral small vessel disease, *n* (%)	1045 (31.1)	90 (33.8)	786 (30.9)	169 (30.8)	.604

Abbreviations: SD, standard deviation; SEP, socioeconomic position; T2DM, type 2 diabetes mellitus; LIBRA1, the original LIfestyle for BRAin health score.

^*^The SEP compound score was calculated using the mean of *z*-scores for education, occupation, and household income, categorized into tertiles: 1 = low, 2 = middle, 3 = high.

^†^TIB ranged from 4.66 to 12.66 h/night.

^‡^Sleep breaks ranged from 0 to 13 interruptions per night.

§ Based on *n* = 3333 due to missing values.

|| Based on *n* = 3331 due to missing values.

### Objective sleep parameters

#### Time in bed

**Figure 2:**
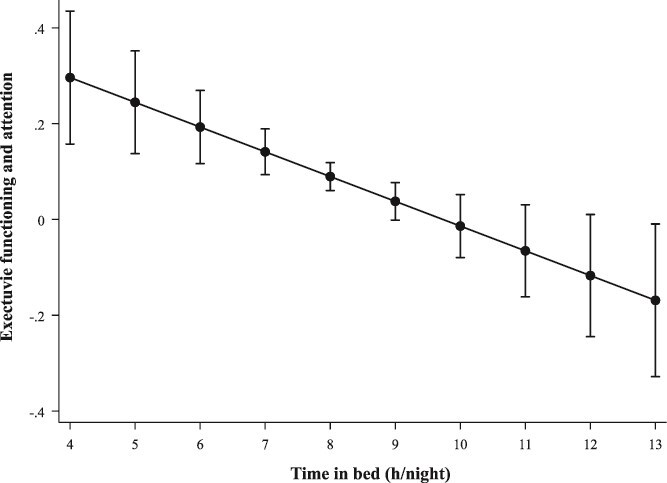
Estimated marginal means depicting the association between TIB (h/night) and executive functioning and attention. Adjusted for age, sex, composite SEP score, original LIfestyle for BRAin health (LIBRA1) score, and sleep medication usage (Model 3).

After fully adjusting for demographic factors, clinical and lifestyle factors and sleep medication usage (Model 3), a significant negative linear association was observed between TIB (h/night) and executive functioning and attention (B_linear_ = −0.052, 95% CI = −0.084 to −0.019, *p* = .002); see Table 2. Figure 2 shows that increasing TIB was associated with lower executive functioning and attention scores. However, no significant associations were found between TIB and overall cognition, memory, or information processing speed.

**Table 2:** Associations between Objective TIB and Cognitive Functioning

	Time in bed (h/night) (continuous)		Time in bed (categories^*^)			
	Linear term		Short (<7 h/night)		Long (≥9 h/night)	
	B [95% CI]	*p*	B [95% CI]	*p*	B [95% CI]	*p*
***Overall cognition***						
Crude model	**−0.035 [−0.059, −0.011]**	**.005**	0.006 [−0.073, 0.086]	.881	**−0.087 [−0.145, −0.029]**	**.003**
Model 1	**−0.019 [−0.038, −0.000]**	**.049**	0.017 [−0.046, 0.079]	.599	−0.035 [−0.080, 0.011]	.137
Model 2	−0.017 [−0.036, 0.002]	.075	0.014 [−0.048, 0.076]	.661	−0.032 [−0.078, 0.013]	.169
Model 3	−0.017 [−0.036, 0.002]	.080	0.014 [−0.048, 0.076]	.662	−0.032 [−0.078, 0.013]	.167
***Memory***						
Crude model	−0.008 [−0.045, 0.028]	.648	0.005 [−0.115, 0.126]	.929	−0.019 [−0.107, 0.069]	.665
Model 1	−0.010 [−0.041, 0.022]	.554	0.045 [−0.059, 0.149]	.393	0.010 [−0.066, 0.086]	.799
Model 2	−0.008 [−0.040, 0.023]	.609	0.040 [−0.063, 0.144]	.447	0.011 [−0.065, 0.087]	.782
Model 3	−0.008 [−0.040, 0.023]	.615	0.040 [−0.064, 0.144]	.447	0.011 [−0.065, 0.087]	.783
***Information processing speed***						
Crude model	−0.030 [−0.066, 0.005]	.091	−0.062 [−0.178, 0.055]	.302	**−0.126 [−0.211, −0.041]**	**.004**
Model 1	−0.005 [−0.034, 0.025]	.763	−0.056 [−0.153, 0.041]	.258	−0.049 [−0.120, 0.023]	.181
Model 2	−0.002 [−0.031, 0.028]	.900	−0.059 [−0.156, 0.038]	.234	−0.044 [−0.116, 0.027]	.221
Model 3	−0.001 [−0.031, 0.028]	.934	−0.059 [−0.156, 0.038]	.233	−0.045 [−0.116, 0.026]	.218
***Executive functioning and attention***						
Crude model	**−0.088 [−0.124. −0.052]**	**<.001**	0.076 [−0.044, 0.195]	.215	**−0.177 [−0.264, −0.089]**	**<.001**
Model 1	**−0.054 [−0.087, −0.022]**	**.001**	0.063 [−0.044, 0.170]	.249	**−0.092 [−0.170, −0.014]**	**.021**
Model 2	**−0.052 [−0.085, −0.020]**	**.002**	0.061 [−0.046, 0.168]	.262	**−0.088 [−0.166, −0.010]**	**.028**
Model 3	**−0.052 [−0.084, −0.019]**	**.002**	0.061 [−0.046, 0.168]	.262	**−0.088 [−0.166, −0.010]**	**.028**

Abbreviations: CI, confidence interval; B, unstandardized regression coefficient.

^*^A mid-range (≥7 to <9 h/night) TIB served as a reference group.

Crude model: no covariates.

Model 1: analyses adjusted for age, sex, composite SEP score, T2DM.

Model 2: Model 1 + original LIfestyle for BRAin health (LIBRA1) score.

Model 3: Model 2 + sleep medication usage.

Bold values indicate statistical significance at *p* < .05.

For the TIB categories (short, mid-range, or long), having a long TIB (≥9 h/night) was significantly associated with lower executive functioning and attention scores (B = −0.088, 95% CI = −0.166 to −0.010, *p* = .028) compared to the reference group with a mid-range TIB (≥7–<9 h/night) (Table 2). No other statistically significant associations were identified between TIB categories and overall cognition, memory, or information processing speed.

**Figure 3:**
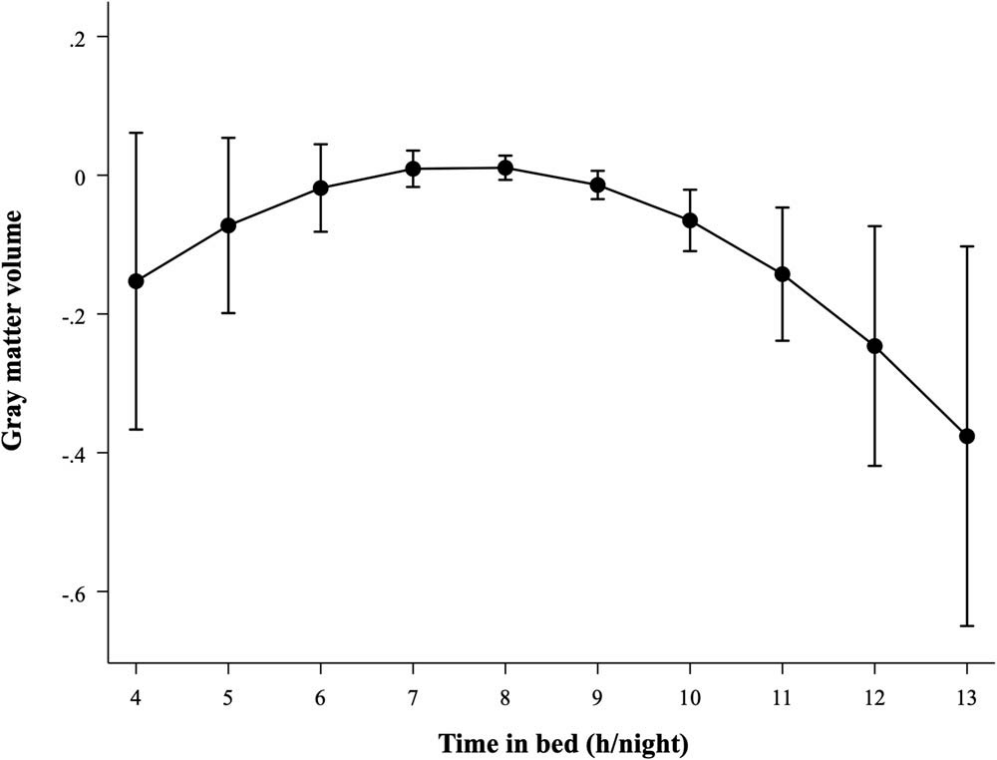
Estimated marginal means depicting the association between TIB (h/night) and GM volume. Adjusted for MRI lag time, ICV, age, sex, composite SEP score, original LIfestyle for BRAin health (LIBRA1) score, and sleep medication usage (Model 3).

An LR test indicated that a curvilinear association better represented the association between TIB and GM volume compared to a linear association (LR: *χ*^2^ [1] = 4.8, *p* = .029). After adjustments for demographic factors, clinical and lifestyle factors, and sleep medication usage (Model 3), a curvilinear association was observed between TIB and lower GM volume (B_linear_ = −0.017, 95% CI = −0.034 to −0.001, *p* = .043; B_quadratic_ = −0.013, 95% CI = −0.025 to −0.001, *p* = .029). Predicted GM volumes were lower at both shorter (6 h/night: −0.018) and longer (10 h/night: −0.065) TIB durations compared to 8 h/night (0.011), indicating an inverted U-shaped association as illustrated in Figure 3. No significant associations were found in the TIB categories analyses (Table 3).

**Table 3:** Associations between Objective Sleep Parameters and Markers of Brain Morphology and CSVD

	Time in bed (h/night) (continuous)		Time in bed (categories^*^)			
			Short (<7 h/night)		Long (≥9 h/night)	
Brain morphology marker^†^	B [95% CI]	*p*	B [95% CI]	*p*	B [95% CI]	*p*
***Gray matter volume***						
Crude Model	**−0.030 [−0.049, −0.011]**	**.002**	0.010 [−0.052, 0.073]	.747	**−0.068 [−0.114, −0.022]**	**.004**
Model 1	**−0.020 [−0.037, −0.003]**	**.020**	0.012 [−0.044, 0.068]	.684	−0.038 [−0.079, 0.002]	.066
Model 2	**−0.017 [−0.034, −0.002]**	**.047**	0.010 [−0.045, 0.066]	.720	−0.033 [−0.074, 0.008]	.112
Model 3	**−0.017 [−0.034, −0.001]**	**.043**	0.010 [−0.045, 0.066]	.719	−0.033 [−0.074, 0.008]	.114
Model 3 (quadratic term)^‡^	**−0.013 [−0.025, −0.001]**	**.029**	–	–	–	–
***White matter volume***						
Crude Model	−0.005 [−0.023, 0.013]	.581	0.008 [−0.052, 0.067]	.800	0.000 [−0.043. 0.044]	.994
Model 1	−0.000 [−0.017, 0.018]	.960	0.010 [−0.048, 0.067]	.739	0.015 [−0.027, 0.057]	.485
Model 2	0.000 [−0.017, 0.018]	.978	0.005 [−0.053, 0.063]	.863	0.015 [−0.027, 0.057]	.482
Model 3	0.001 [−0.017, 0.018]	.937	0.005 [−0.053, 0.063]	.864	0.015 [−0.027, 0.057]	.485
***Right hippocampal volume***						
Crude Mode	−0.028 [−0.058, 0.002]	.064	−0.009 [−0.108, 0.089]	.855	−0.072 [−0.144, 0.000]	.051
Model 1	−0.014 [−0.042, 0.014]	.313	−0.015 [−0.107, 0.077]	.754	−0.034 [−0.101, 0.034]	.324
Model 2	−0.013 [−0.041, 0.015]	.349	−0.020 [−0.112, 0.072]	.672	−0.034 [−0.101, 0.034]	.329
Model 3	−0.013 [−0.041, 0.015]	.355	−0.019 [−0.112, 0.072]	.672	−0.034 [−0.101, 0.034]	.328
***Left hippocampal volume***						
Crude Model	−0.020 [−0.050, 0.010]	.186	0.001 [−0.097, 0.100]	.983	−0.041 [−0.113, 0.031]	.270
Model 1	−0.006 [−0.034, 0.022]	.681	−0.007 [−0.098, 0.085]	.888	−0.002 [−0.069, 0.065]	.958
Model 2	−0.005 [−0.032, 0.023]	.738	−0.010 [−0.102, 0.081]	.825	−0.001 [−0.068, 0.066]	.978
Model 3	−0.005 [−0.032, 0.023]	.745	−0.010 [−0.102, 0.081]	.825	−0.001 [−0.067, 0.066]	.977
***Total hippocampal volume***						
Crude Model	−0.025 [−0.054, 0.005]	.099	−0.004 [−0.101, 0.093]	.933	−0.058 [−0.129, 0.014]	.113
Model 1	−0.010 [−0.038, 0.017]	.457	−0.011 [−0.101, 0.079]	.812	−0.018 [−0.084, 0.047]	.585
Model 2	−0.009 [−0.036, 0.018]	.506	−0.016 [−0.106, 0.075]	.736	−0.018 [−0.084, 0.048]	.596
Model 3	−0.009 [−0.036, 0.018]	.512	−0.015 [−0.105, 0.074]	.736	−0.018 [−0.084, 0.048]	.596
***White matter hyperintensity volume***						
Crude Model	0.017 [−0.019, 0.053]	.360	0.011 [−0.108, 0.130]	.862	0.034 [−0.053, 0.121]	.447
Model 1	−0.008 [−0.041, 0.025]	.629	0.022 [−0.087, 0.130]	.693	−0.029 [−0.108, 0.051]	.481
Model 2	−0.012 [−0.046, 0.020]	.482	0.029 [−0.079, 0.138]	.594	−0.035 [−0.114, 0.045]	.394
Model 3	−0.012 [−0.045, 0.021]	.465	0.029 [−0.079, 0.138]	.595	−0.035 [−0.114, 0.045]	.392
***Cerebrospinal fluid volume***						
Crude Model	**0.042 [0.014, 0.071]**	**.004**	−0.020 [−0.115, 0.074]	.670	**0.083 [0.014, 0.151]**	**.019**
Model 1	**0.026 [0.002, 0.049]**	**.033**	−0.025 [−0.103, 0.053]	.533	0.030 [−0.027, 0.087]	.304
Model 2	0.021 [−0.003, 0.045]	.082	−0.017 [−0.096, 0.060]	.655	0.023 [−0.034, 0.080]	.429
Model 3	0.021 [−0.003, 0.045]	.080	−0.018 [−0.096, 0.060]	.654	0.023 [−0.034, 0.080]	.430
***Presence of cerebral small vessel disease (yes/no)***						
Crude Model	OR: 1.010 [0.929, 1.096]	.831	OR: 1.153 [0.882, 1.508]	.297	OR: 0.993 [0.813, 1.214]	.948
Model 1	OR: 0.955 [0.874, 1.042]	.301	OR: 1.238 [0.931, 1.648]	.142	OR: 0.872 [0.704, 1.080]	.208
Model 2	OR: 0.950 [0.870, 1.038]	.243	OR: 1.245 [0.936, 1.657]	.133	OR: 0.865 [0.698, 1.070]	.182
Model 3	OR: 0.950 [0.870, 1.037]	.249	OR: 1.245 [0.936, 1.658]	.132	OR: 0.864 [0.698, 1.070]	.179

Abbreviations: CI, confidence interval; B, unstandardized regression coefficient; OR, odds ratio.

^*^A mid-range (≥7 to <9 h/night) TIB served as a reference group.

^†^Standardized brain volume (*z*-scores).

^‡^A quadratic term for TIB was additionally included, demonstrating a better fit than the simpler Model 3, which used only the linear term.

Crude model: adjusted for MRI lag time and ICV for volumetric MRI markers.

Model 1: analyses adjusted for age, sex, composite SEP score, T2DM.

Model 2: Model 1 + original LIfestyle for BRAin health (LIBRA1) score.

Model 3: Model 2 + sleep medication usage.

Bold values indicate statistical significance at *p* < .05.

Upon full adjustment for covariates in Model 3, no associations emerged between TIB and volumetric markers of WM, right, left, and total hippocampal volume, CSF, WMH, and the presence of CSVD. Also, no significant associations were observed for markers of brain morphology across the TIB categories (Table 3).

#### Sleep breaks

**Figure 4:**
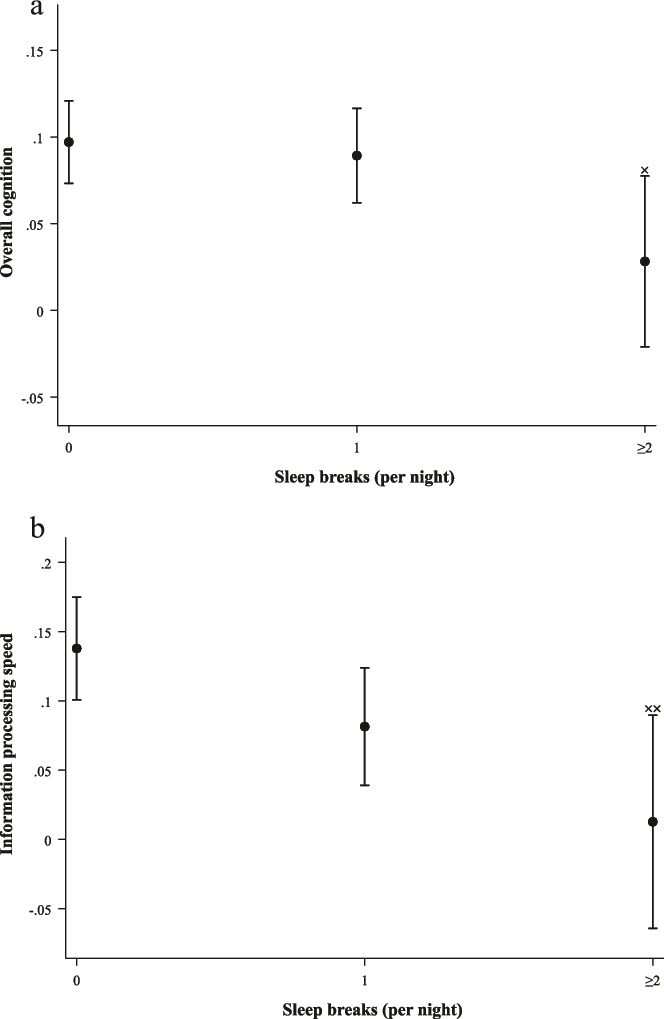
(a and b) Estimated marginal means depicting the association between sleep breaks (per night) and (a) overall cognition; (b) information processing speed. (a) Adjusted for age, sex, composite SEP score, original LIfestyle for BRAin health (LIBRA1) score, TIB, and sleep medication usage (Model 3); ^x^*p* < .05. (b) Adjusted for age, sex, composite SEP score, original LIfestyle for BRAin health (LIBRA1) score, TIB, and sleep medication usage (Model 3); ^xx^*p* < .01.

After adjusting for demographic factors, clinical and lifestyle factors, sleep medication usage, and TIB, sleep breaks (≥2/night) were significantly associated with worse overall cognition (B = −0.069, 95% CI = −0.124 to −0.013, *p* = .015) (Figure 4, A) and slower information processing speed (B = −0.125, 95% CI = −0.212 to −0.039, *p* = .005) when compared to the reference group with no sleep breaks (Figure 4, B). No significant associations were found for memory and executive functioning and attention for participants who exhibited 1 or ≥2 sleep breaks per night (Supplementary Table S4).

**Figure 5:**
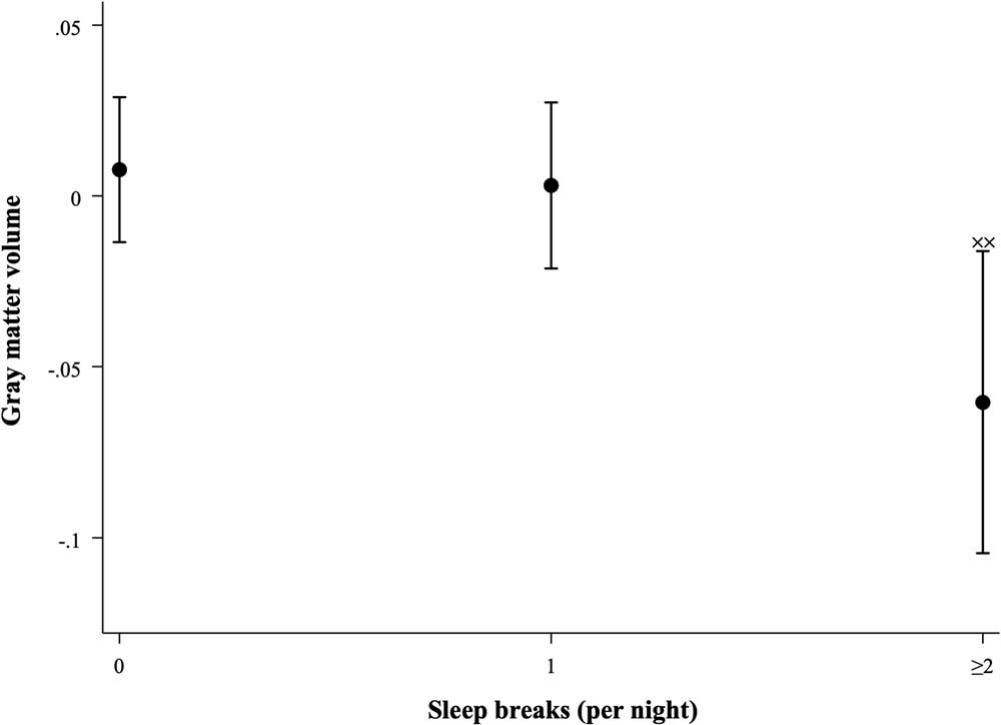
Estimated marginal means depicting the association between sleep breaks (per night) and GM volume. Adjusted for MRI lag time, ICV, age, sex, composite SEP score, original LIfestyle for BRAin health (LIBRA1) score, TIB, and sleep medication usage (Model 3); ^xx^*p* < .01.

For sleep breaks, a significant negative association was observed between those with ≥2 sleep breaks and GM volume (B = −0.068, 95% CI = −0.118 to −0.018, *p* = .007) compared to those with no sleep breaks (Figure 5), but not for individuals with only one sleep interruption at night. No significant associations were found between sleep breaks and other volumetric markers or CSVD (Supplementary Table S5).

### Subjective sleep measures

#### Sleep continuity

After adjusting for demographic factors, clinical and lifestyle factors, and sleep medication usage in Model 3, individuals reporting sleep continuity did not exhibit better overall cognition or functioning in specific cognitive domains compared to those reporting sleep discontinuity (Supplementary Table S6). Furthermore, no associations were found between sleep continuity and any volumetric markers or CSVD (Supplementary Table S7).

#### Excessive daytime sleepiness

Excessive daytime sleepiness (≥11 points) was not associated with worse cognitive functioning, volumetric markers, or CSV after adjustments for covariates in Model 3 (Supplementary Tables S6 and S7).

### Potential effect modifiers

The association between executive functioning and attention and TIB was moderated by age (B_linear_ = 0.004, 95% CI = 0.000 to 0.008 *p* = .035). Stratified analyses indicated among individuals aged 60 years and younger, a longer TIB was associated with worse executive functioning and attention scores (B_linear ≤ 60 years_ = −0.078, 95% CI = −0.123 to −0.032, *p* = .001; B_linear > 60 years_ = −0.013, 95% CI = −0.061 to 0.036, *p* = .607). Additionally, the association between GM volume and sleep breaks (≥2/night) was moderated by sex (B_linear_ = 0.022, 95% CI = 0.017 to 0.221, *p* = .022). Further analyses showed males with sleep breaks had lower GM volumes compared to males with no sleep breaks (B_1 interruption per night_ = 0.010, 95% CI = −0.042 to 0.062, *p* = .704; B_≥2 interruptions per night_ = −0.076, 95% CI = −0.147 to −0.005, *p* = .035). There was a positive interaction between the second composite SEP tertile score and TIB (quadratic term) in relation to GM volume (B_linear_ = 0.050, 95% CI = 0.010 to 0.091, *p* = .015); B_quadratic_ = 0.003, 95% CI = 0.001 to 0.005, *p* = .016). However, stratified analyses by composite SEP tertile did not reveal significant associations between TIB and GM volume within any tertile group (B_quadratic in first composite SEP tertile score_ = −0.013, 95% CI = −0.033 to 0.006, *p* = .170; B_quadratic in second composite SEP tertile score_ = −0.016, 95% CI = −0.037 to 0.005, *p* = .132; B_quadratic in third composite SEP tertile score_ = −0.009, 95% CI = −0.032 to 0.013, *p* = .403). No other significant interactions for age, sex, T2DM, and composite SEP score were found.

### Sensitivity analyses

Sensitivity analyses were conducted to investigate associations between sleep parameters and cognitive functioning using Model 3, including participants regardless of whether they had MRI data available (*n* = 5042). Most findings remained unchanged (Supplementary Tables S8–S10). TIB retained its negative association with executive functioning and attention. Similarly, the negative association between sleep breaks (≥2/night) and overall cognition and information processing speed remained. Subjective sleep parameters continued to show no significant associations with cognitive functioning. New findings included a negative linear association between TIB and overall cognition speed (B_linear_ = −0.018, 95% CI = −0.033 to −0.003, *p* = .022). Specifically individuals with a longer TIB (≥9 h/night) had worse overall cognition (B = −0.041, 95% CI = −0.079 to −0.004, *p* = .032). Moreover, a curvilinear association was observed between TIB and information processing speed (B_quadratic_ = −0.020, 95% CI = −0.036 to −0.003, *p* = .018). Regarding sleep breaks, having even one sleep break per night was also associated with worse information processing speed (B = −0.051, 95% CI = −0.098 to −0.003, *p* = .036), while having ≥2/night was associated with worse executive functioning and attention (B = −0.104, 95% CI = −0.173 to −0.034, *p* = .003). These additional results likely reflect the increased statistical power due to an increased sample size and lower potential selection bias in the more complete dataset. Participants excluded from the main analyses due to missing MRI data were older, had lower composite SEP scores, higher mean LIBRA1 scores, poorer cognitive function, and worse sleep outcomes.

Another sensitivity analysis was conducted in which the SEP components—educational attainment, household income, and occupational status—were included separately in the main model (Model 3). Overall, findings remained largely consistent; however, some differences did emerge, which may be attributed to differences in sample size (*n* = 2459 vs *n* = 3360) (see Supplementary Text S1).

## Discussion

In this community-based study of adults aged 40 to 75, TIB was negatively associated with executive functioning and attention, even after adjusting for demographic, clinical, lifestyle-related factors, and sleep medication. Specifically, those with a long TIB (≥9 h/night) exhibited poorer executive functioning and attention, with no significant differences observed in other cognitive domains. TIB also exhibited an inverted U-shaped relationship with GM volume, with individuals sleeping ≥7 to <9 h showing higher GM. However, no significant associations were found between TIB and other morphological brain markers. Additionally, increased sleep breaks were associated with worse overall cognition, slower information processing speed, and lower GM volume. In contrast, subjective sleep parameters, such as sleep continuity and excessive daytime sleepiness, showed no significant associations with cognitive functioning or markers of brain morphology.

### Time in bed

Individuals with a longer TIB showed poorer executive functioning and attention scores, particularly among those aged 60 years or younger. Specifically, those categorized as having a long TIB (≥9 h/night) demonstrated significantly poorer performance in this cognitive domain compared to those with a mid-range TIB (≥7 to <9 h/night). This finding is consistent with a previous population-based cohort study involving adults aged 60 years or older, which used subjective TIB calculated from the Pittsburgh Sleep Quality Index. The study found that a longer TIB (>9 h) was associated with a greater decline in Mini-Mental State Examination score; however, this association was primarily observed in men or participants aged 60–74 years [12]. In contrast, a meta-analysis of 33 studies using accelerometry or polysomnography found no consistent associations between objectively measured total sleep time or TIB and cognitive domains [28]. The reason for the association between TIB and only executive function and attention in the current study remains unclear, especially given that few studies have directly examined TIB in relation to cognition. In individuals under 60, this association may be influenced by factors such as work-related demands, family and social obligations, and stress, all of which can lead to sleep disturbances. Thus, a longer TIB may reflect fatigue or poor sleep quality, potentially impairing executive function and attention. Nonetheless, sensitivity analyses in a larger sample of participants identified additional associations with information processing speed and overall cognition. An underestimation of associations may have occurred in the main analyses due to a “healthy participant effect.”

Furthermore, participants with both a shorter and longer TIB demonstrated lower GM volume. In the categorical analyses, although the differences between individuals with short (<7 h/night) and long (≥9 h/night) TIB, compared to those with a mid-range TIB (≥7 to <9 h/night), were not statistically significant, the estimates were stronger among individuals with a long TIB. It is also important to note that data on individuals with a short TIB were scarcer with wider CIs. Due to the scarcity of objective sleep studies assessing parameters such as TIB in relation to brain morphology, direct comparisons with previous studies remain challenging. The MIND-China study, for example, reported associations with GM volume among long sleepers (>8 h of sleep duration), as measured using electrocardiogram-based cardiopulmonary coupling analysis, with no significant links to other morphological markers [56]. Additionally, other brain morphological markers, such as WM volume and WMH, were not associated with TIB in the current study. In contrast, a longitudinal study found that wrist-worn accelerometry-measured shorter sleep duration was associated with poorer WM microstructure over time, although other morphological markers were not examined [57]. The current, limited, and inconsistent evidence highlights the importance of clarifying how TIB and sleep duration are independently associated with brain morphology.

TIB, although distinct from sleep duration, may reflect both sleep opportunity [11] and underlying sleep disturbances [58], with implications for cognitive functioning and brain morphology. Sufficient sleep is needed to cycle through to nonrapid eye movement slow wave sleep, during which the glymphatic system is exclusively active [59]. Thus, a shorter TIB may lead to reduced sleep durations and hinder glymphatic drainage, impeding the removal of metabolic waste products like β-amyloid and tau protein, the pathological hallmarks of Alzheimer’s disease and dementia [59–61]. The accumulation of neurotoxic metabolites can instigate the breakdown of the blood–brain barrier, leading to an increase in neuroinflammation and neuronal injury, thereby exacerbating general cognitive decline [62] and neurodegeneration [63]. In contrast, the mechanisms underlying the effects of a long TIB remain incompletely understood. A prolonged TIB may be a symptom of underlying health issues, such as fatigue or disrupted sleep patterns, which could be associated with conditions like sleep disorders. For example, previous research has linked obstructive sleep apnoea with changes in brain regions, such as increased WMH [64], GM volume loss [65], and cognitive impairment [66]. Furthermore, a longer TIB (as well as a shorter TIB) may be a consequence of depression, a recognized risk factor for dementia [3], though the current study controlled for depression in its analyses. Additionally, a longer TIB may be associated with higher levels of inflammatory markers, including C-reactive protein, interleukin-6, and tumor necrosis factor [67], which can negatively impact cognitive functioning [68].

### Sleep breaks

Sleep breaks are an important determinant of perceived sleep quality [69]. They are linked to several lifestyle factors, such as caffeine, alcohol, heavy meals, and light exposure shortly before bedtime [70], and conditions such as nocturia [71]. The current study reveals that participants with more sleep breaks (≥2/night) had worse overall cognition, slower information processing speed, and lower GM volume. These results are supported by previous literature using objectively measured sleep breaks [19, 72–75]. It has been proposed that sleep breaks are linked to lower CSF β-amyloid 42 levels, which indicate greater accumulation of β-amyloid in the brain [76] and increased neuroinflammation through the activation and aging of microglia [77]. Notably, lower CSF β-amyloid 42 levels have been associated with GM atrophy in the parietal, temporal, and frontal regions, as well as left hippocampal atrophy [78], providing a plausible neurobiological mechanism by which sleep breaks may contribute to structural brain changes. Sleep-related breathing disorders, with males at a higher risk than females [79], can also lead to recurrent sleep arousals and blood oxygen desaturation, which are associated with lower GM volume in the frontal pole- a region proposed to integrate cognitive processes across multiple domains- and lower cognitive performance [80]. In the current study, males with sleep breaks (≥2 interruptions/night) had lower GM volumes, potentially due to the presence of a sleep-related breathing disorder; however, this remains purely speculative. This emphasizes the importance of including sleep breaks in research alongside TIB and highlights the need to account for its underlying causes, such as lifestyle factors and sleep-related breathing disorders.

### Subjective sleep continuity and excessive daytime sleepiness

In contrast, self-reported sleep continuity was not associated with cognitive functioning or markers of brain morphology. These findings align with The Rush Memory and Aging Project, which examined whether greater sleep breaks are associated with regionally decreased GM volume in older community-dwelling adults. The study reported that self-reported sleep continuity, assessed by the question “How often are you troubled by waking up in the night?” on an ordinal scale from 0 (“never”) to 5 (“very often”), was not associated with cortical GM volume [19]. On the other hand, findings from The Gothenburg H70 Birth Cohort Studies showed that participants who reported waking up once or multiple times at night had worse verbal fluency and global cognition, even after adjusting for demographic factors, lifestyle factors, hypnotics, depressive symptoms, and cardiovascular health [81]. One potential explanation for these conflicting findings is that sleep continuity in the current study was assessed rather broadly, using a single-item binary question, which may have limited sensitivity. As a result, responses may have been influenced by individual beliefs, expectations, and cognitive status. This contrasts with other studies that employed ordinal scales [19] or more detailed questions [81], allowing for a more nuanced assessment of sleep continuity. In addition, a discrepancy in the current study was observed between objective sleep breaks and subjective sleep continuity, which may underscore the limitations of relying solely on subjective measures to assess sleep continuity.

Similarly, excessive daytime sleepiness in this study did not exhibit significant associations with cognitive functioning or markers of brain morphology, contrary to findings in previous literature [9]. A meta-analysis, including 14 observational cohort studies, revealed that excessive daytime sleepiness was linked to an increase in all-cause cognitive decline or dementia [9]. However, it should be highlighted that the studies utilized different definitions of excessive daytime sleepiness, ranging from the ESS to the Pittsburgh Sleep Quality Index and the Medical Outcomes Study Sleep Scale, as well as study-specific questions, which may have potentially distorted effect sizes. Moreover, results from the UK Biobank cohort suggested that excessive daytime sleepiness was associated with reduced GM volume in regions such as the right paracingulate gyrus and the left occipital pole [82], as well as increased WMH [83]. The lack of significant results in the current study may be attributed to the study sample being relatively healthy, which could allow for brain maintenance and functional compensation [84] to mitigate the effects of excessive daytime sleepiness. Additionally, important confounders such as obstructive sleep apnoea and napping may have obscured potential effects. However, relevant data on these variables were not available in the present study.

### Strengths and limitations

This study has several notable strengths, such as a large sample size and a population-based design. The Maastricht Study’s extensive phenotyping approach enabled the assessment of cognitive functioning across multiple domains, along with MRI scans, which is a distinctive feature of this study. Additionally, a wide range of covariates were considered, such as sleep medication usage and 11 risk and protective factors for dementia. Relevant confounders, including depression, physical inactivity, cardiovascular risk factors, and alcohol intake, were also accounted for within the LIBRA1 health index.

However, this study has limitations that should be acknowledged. While the use of accelerometry in this study offers advantages such as recording TIB over multiple days in large samples, being less burdensome, and providing greater ecological validity than subjective measures [85], some drawbacks remain. Thigh-worn accelerometry is less commonly used in sleep research compared to wrist-worn devices [86, 87]. However, validation studies comparing thigh-worn accelerometers with polysomnography have shown similar results to those observed in the wrist-worn validation studies [88]. Moreover, accelerometers measure acceleration and, therefore, cannot distinguish between physiological sleep and periods of wakefulness, potentially leading to the misclassification of sleep [89]. In addition, sleep breaks were measured based on changes in thigh movement, and not all sleep interruptions necessarily result in such movements [88]. The current study operationalized sleep breaks as the number of sleep interruptions per night, whereas other studies have used different parameters, such as wake after sleep onset, nighttime motor restlessness, or sleep efficiency [28], which may affect comparability across research. Furthermore, the cross-sectional design of the study limits the ability to infer causality, which is particularly relevant in the context of potential reverse causation. Indeed, a bi-directional relationship between sleep, cognition [90], and brain morphology [91, 92] has been previously reported. For example, neurodegenerative changes preceding the development of dementia may contribute to sleep disturbances, rather than cause them [93]. Adding to this, the current study’s sample was predominantly Caucasian. Research has shown that sleep disturbances can differ across racial groups [94]; thus, the findings may not be representative of other populations. Lastly, although pre-specified covariates were adjusted for using a systematic, literature-informed stepwise approach rather than multiple testing, the possibility of a type I error remains. Therefore, the results should be interpreted with caution.

### Clinical implications and future recommendations

The current study’s findings contribute to the growing evidence of sleep’s significant role in cognitive functioning and brain morphology, even when considering other factors linked to brain health, as summarized in the LIBRA1 score [7]. Given the high prevalence of sleep disorders and insufficient sleep within the Dutch population [95], as well as the study’s findings that a long TIB (≥9 h), in particular, may be related to adverse effects on cognitive function and brain morphology, public health initiatives focused on improving sleep hygiene are essential. Strategies like sleep diplomacy, an approach that emphasizes the importance of promoting better sleep practices in the general population, should be actively encouraged [96].

Future studies are needed to replicate and validate these findings using a longitudinal approach with extensive follow-ups. In addition to the sleep parameters already investigated, healthy sleep encompasses several other components, such as chronotype, regularity, and sleep disorders, which should also be explored [97, 98]. Moreover, future studies should go beyond examining global brain volumes and investigate distinct regions, such as the thalamus and the brainstem, which are known to be involved in sleep physiology [99]. Additionally, the role of brain connectivity warrants further exploration, particularly in light of evidence that sleep deprivation impacts the brain networks essential for executive functions [100].

## Conclusion

This study found that a longer TIB was cross-sectionally related to worse executive functioning and attention, as well as with both a shorter and a longer TIB duration being associated with lower GM volume. Additionally, increased sleep breaks were linked to poorer cognitive performance and reduced GM volume. In contrast, subjective sleep parameters showed no significant associations with cognitive functioning or brain morphology. These results underscore the potential importance of adequate and uninterrupted TIB for preserving cognitive function and brain health. They also highlight the need for future research to further explore longitudinal associations and other aspects of sleep, which are essential for informing targeted interventions to improve sleep health and support healthy brain aging.

## Supplementary Material

Supplementary_Materials_zsaf218

## Data Availability

The data of this study derive from The Maastricht Study, but restrictions apply to the availability of data, which were used under license for the current study. Data is, however, available from the authors upon reasonable request and with permission of The Maastricht Study management team.
