# Supplementary material for: The association between sleep parameters, cognitive functioning, and markers of brain morphology: The Maastricht Study
Source: Sleep. 2025 Jul 28;49(3):zsaf218. doi: 10.1093/sleep/zsaf218 (PMC13016686; doi:10.1093/sleep/zsaf218)
Supplement: Supplementary_Materials_zsaf218 [file supplementary_materials_zsaf218.docx]

**The association between sleep parameters, cognitive functioning, and markers of brain morphology: *The Maastricht Study***

Tessa L. van Baal^1,2^, Sebastian Köhler^1,2^, Annemarie Koster^3,4^, Ree M. Meertens^3,5,6^, Hans Bosma^3,4^, Miranda T. Schram^1,7,8,9,10^, Jacobus F. A. Jansen^1,11,12^, Sami O. Simons^5,13^, Bastiaan E. de Galan^7,8,14^, Simone J. P. M. Eussen^3,8,15^, Niels Janssen^1,2^, Kay Deckers^1,2^

^1^ Mental Health and Neuroscience Research Institute (MHeNs), Maastricht University, Maastricht, The Netherlands.

^2^ Alzheimer Centre Limburg, Department of Psychiatry and Neuropsychology, Maastricht University, Maastricht, The Netherlands.

^3^ Care and Public Health Research Institute (CAPHRI), Maastricht University, Maastricht, The Netherlands.

^4^ Department of Social Medicine, Maastricht University, Maastricht, The Netherlands.

^5^ Institute of Nutrition and Translational Research in Metabolism (NUTRIM), Maastricht University, Maastricht, The Netherlands.

^6^ Department of Health Promotion, Maastricht University, Maastricht, The Netherlands.

^7^ Department of Internal Medicine, Maastricht University Medical Centre+, Maastricht, The Netherlands.

^8^ Cardiovascular Research Institute Maastricht (CARIM), Maastricht University, Maastricht, The Netherlands.

^9^ Heart and Vascular Centre, Maastricht University Medical Centre+, Maastricht, The Netherlands.

^10^ Department of Epidemiology, Erasmus Medical Centre, Rotterdam, The Netherlands.

^11^ Department of Radiology and Nuclear Medicine, Maastricht University Medical Centre+, The Netherlands.

^12^ Department of Electrical Engineering, Eindhoven University of Technology, Eindhoven, The Netherlands.

^13^ Department of Respiratory Medicine, Maastricht University Medical Centre+, The Netherlands.

^14^ Department of Internal Medicine, Radboud University Medical Centre, Nijmegen, The Netherlands.

^15^Department of Epidemiology, Maastricht University, Maastricht, The Netherlands.

**Correspondence to:** Kay Deckers, PhD, Alzheimer Centre Limburg, Mental Health and Neuroscience Research Institute, Maastricht University, PO Box 616, 6200 MD, Maastricht, The Netherlands; T: 0031-43-3384104; E: [kay.deckers@maastrichtuniversity.nl](mailto:kay.deckers@maastrichtuniversity.nl)

**SUPPLEMENTARY TABLES**

**Table S1.** STROBE Statement—checklist of items that should be included in reports of observational studies.

|  | **Item No.** | **Recommendation** | **Page**  **No.** |
| --- | --- | --- | --- |
| Title and abstract | 1 | (*a*) Indicate the study’s design with a commonly used term in the title or the abstract | 1 |
|  |  | (*b*) Provide in the abstract an informative and balanced summary of what was done and what was found | 2-3 |
| Introduction | | |  |
| Background/rationale | 2 | Explain the scientific background and rationale for the investigation being reported | 5-7 |
| Objectives | 3 | State specific objectives, including any prespecified hypotheses | 7 |
| Methods | | |  |
| Study design | 4 | Present key elements of study design early in the paper | 8 |
| Setting | 5 | Describe the setting, locations, and relevant dates, including periods of recruitment, exposure, follow-up, and data collection | 8 |
| Participants | 6 | (*a*) *Cohort study*—Give the eligibility criteria, and the sources and methods of selection of participants. Describe methods of follow-up  *Case-control study*—Give the eligibility criteria, and the sources and methods of case ascertainment and control selection. Give the rationale for the choice of cases and controls  *Cross-sectional study*—Give the eligibility criteria, and the sources and methods of selection of participants | 8 |
|  |  | (*b*) *Cohort study*—For matched studies, give matching criteria and number of exposed and unexposed  *Case-control study*—For matched studies, give matching criteria and the number of controls per case |  |
| Variables | 7 | Clearly define all outcomes, exposures, predictors, potential confounders, and effect modifiers. Give diagnostic criteria, if applicable | 9-13 |
| Data sources/ measurement | 8* | For each variable of interest, give sources of data and details of methods of assessment (measurement). Describe comparability of assessment methods if there is more than one group | 9-13 |
| Bias | 9 | Describe any efforts to address potential sources of bias | 14-15 |
| Study size | 10 | Explain how the study size was arrived at | 8 |
| Quantitative variables | 11 | Explain how quantitative variables were handled in the analyses. If applicable, describe which groupings were chosen and why | 13-15 |
| Statistical methods | 12 | (*a*) Describe all statistical methods, including those used to control for confounding | 13-15 |
|  |  | (*b*) Describe any methods used to examine subgroups and interactions | 13-15 |
|  |  | (*c*) Explain how missing data were addressed | 13-15 |
|  |  | (*d*) *Cohort study*—If applicable, explain how loss to follow-up was addressed  *Case-control study*—If applicable, explain how matching of cases and controls was addressed  *Cross-sectional study*—If applicable, describe analytical methods taking account of sampling strategy | 13-15 |
|  |  | (*e*) Describe any sensitivity analyses | 15 |

Continued on the next page

| Results | | |  |
| --- | --- | --- | --- |
| Participants | 13* | (a) Report numbers of individuals at each stage of study—e.g. numbers potentially eligible, examined for eligibility, confirmed eligible, included in the study, completing follow-up, and analysed | 16 |
|  |  | (b) Give reasons for non-participation at each stage | 16 |
|  |  | (c) Consider use of a flow diagram | 16 |
| Descriptive data | 14* | (a) Give characteristics of study participants (e.g. demographic, clinical, social) and information on exposures and potential confounders | 16-17 |
|  |  | (b) Indicate number of participants with missing data for each variable of interest | 16 |
|  |  | (c) *Cohort study*—Summarise follow-up time (e.g., average and total amount) |  |
| Outcome data | 15* | *Cohort study*—Report numbers of outcome events or summary measures over time |  |
|  |  | *Case-control study—*Report numbers in each exposure category, or summary measures of exposure |  |
|  |  | *Cross-sectional study—*Report numbers of outcome events or summary measures | 17 |
| Main results | 16 | (*a*) Give unadjusted estimates and, if applicable, confounder-adjusted estimates and their precision (eg, 95% confidence interval). Make clear which confounders were adjusted for and why they were included | 17-20 |
|  |  | (*b*) Report category boundaries when continuous variables were categorized | 17-20 |
|  |  | (*c*) If relevant, consider translating estimates of relative risk into absolute risk for a meaningful time period |  |
| Other analyses | 17 | Report other analyses done—e.g. analyses of subgroups and interactions, and sensitivity analyses | 20-22 |
| Discussion | | |  |
| Key results | 18 | Summarise key results with reference to study objectives | 23 |
| Limitations | 19 | Discuss limitations of the study, taking into account sources of potential bias or imprecision. Discuss both direction and magnitude of any potential bias | 28-29 |
| Interpretation | 20 | Give a cautious overall interpretation of results considering objectives, limitations, multiplicity of analyses, results from similar studies, and other relevant evidence | 23-28 |
| Generalisability | 21 | Discuss the generalisability (external validity) of the study results | 29 |
| Other information | | |  |
| Funding | 22 | Give the source of funding and the role of the funders for the present study and, if applicable, for the original study on which the present article is based | 32 |

*Give information separately for cases and controls in case-control studies and, if applicable, for exposed and unexposed groups in cohort and cross-sectional studies.

Note: An Explanation and Elaboration article discusses each checklist item and gives methodological background and published examples of transparent reporting. The STROBE checklist is best used in conjunction with this article (freely available on the Web sites of PLoS Medicine at http://www.plosmedicine.org/, Annals of Internal Medicine at http://www.annals.org/, and Epidemiology at http://www.epidem.com/). Information on the STROBE Initiative is available at www.strobe-statement.org.

**Table S2.** Operationalisation of LIBRA1 factors in The Maastricht Study [1].

| **LIBRA1 factor** | **Weight^a^** | **Operationalised in The Maastricht Study** |
| --- | --- | --- |
| **Adherence to a Mediterranean diet** | -1.7 | Greek Mediterranean diet score (range 0-9) based on a 253-item FFQ (1-year reference period) [2]. Scores ≥6 are categorized as adherence to the diet [3]. |
| **Physical inactivity** | +1.1 | <150 min/week of (self-reported on CHAMPS questionnaire) [4] moderate to vigorous physical activity in the past 2 weeks was categorized as physically inactive [5]. |
| **Smoking** | +1.5 | Self-reported data on smoking cigarettes based on an item of the FFQ. Current smokers were included in the risk score. |
| **Low to moderate alcohol consumption** | -1.0 | Self-reported alcohol intake based on the FFQ. Low to moderate alcohol consumption was defined as <70 gram/week [6]. |
| **Obesity** | +1.6 | BMI^7^ ≥30 kg/m^2^calculated from physical examination at the research centre. |
| **Depression** | +2.1 | Current depressive episode based on the MINI [8] or presence of moderate to severe depressive symptoms based on the PHQ9 (range 0-27; cut-off ≥10) [9]. |
| **Type 2 diabetes** | +1.3 | Glucose tolerance status based on fasting glucose (≥7.0), oral glucose tolerance test (≥11.1), or information on current diabetes medications [10]. |
| **Hypertension** | +1.6 | Average systolic blood pressure ≥140 mmHg, diastolic blood pressure ≥90 mmHg, or current antihypertensive medication use. |
| **Hypercholesterolemia** | +1.4 | Serum total cholesterol ≥6.5mmol/L. |
| **Heart disease** | +1.0 | Self-reported history of cardiovascular disease (cerebrovascular accidents excluded) [11]. |
| **Chronic kidney disease** | +1.1 | Levels of serum cystatin C of <60 and/or average albuminuria categories, based on average urinary albumin excretion. Microalbuminuria and macroalbuminuria were defined as risk [12]. |
| **High cognitive activity** | -3.2 | Data not available in dataset. |
| Abbreviations: BMI = Body Mass Index; CHAMPS = Community Healthy Activities Model Program for Seniors; FFQ = Food Frequency Questionnaire; LIBRA1 = original LIfestyle for BRAin Health; MINI = Mini international Neuropsychiatric Interview; PHQ9 = Patient Health Questionnaire-9.  ^a^Positive weights are assigned to risk factors, and negative weights are assigned to protective factors. Total range -5.9 to 12.7; theoretical range in this cohort: -2.7 to 12.7. | | |

**Table S3.** Participant characteristic differences between the included and excluded sample.

| **Characteristics** | **Included sample**  n= 3,360 | **Excluded sample**  n= 5,827 | **Total sample**  n= 9,187 | **P-value** | **Missing** n |
| --- | --- | --- | --- | --- | --- |
| ***Demographics*** |  |  |  |  |  |
| Age, mean (SD) | 59.5 (8.5) | 59.6 (8.9) | 59.5 (8.8) | 0.886 | 0 |
| Women, n (%) | 1,716 (51.1) | 2,900 (49.8) | 4.616 (50.2) | 0.229 | 0 |
| Composite SEP score^a^, n (%) |  |  |  | <0.001 | 36 |
| Low | 1,018 (30.3) | 2,033 (35.1) | 3,051 (33.2) |  |  |
| Middle | 1,199 (35.7) | 1,853 (32.0) | 3.052 (33.2) |  |  |
| High | 1,143 (34.0) | 1,905 (32.9) | 3,048 (33.2) |  |  |
| T2DM, n (%) | 642 (19.1) | 1,362 (23.4) | 2,004 (21.8) | <0.001 | 3 |
| LIBRA1 score, mean (SD) | 1.2 (2.1) | 1.6 (2.3) * | 1.4 (2.2) | <0.001 | 2,768 |
| Sleep medication, n (%) | 72 (2.1) | 151 (2.6) | 223 (2.4) | 0.173 | 13 |
| ***Objective sleep measures*** |  |  |  |  |  |
| Time in bed (h/night)^b^, mean (SD) | 8.2 (0.9) | 8.2 (1.0) | 8.2 (0.9) | 0.121 | 1,833 |
| Sleep breaks (per night)^c^, n (%) |  |  |  | <0.001 | 1,833 |
| 0 | 1,698 (50.5) | 1,729 (43.3) | 3.427 (37.3) |  |  |
| 1 | 1,267 (37.7) | 1,628 (40.8) | 2,895 (31.5) |  |  |
| ≥2 | 395 (11.8) | 637 (16.0) | 1,032 (11.2) |  |  |
| ***Subjective sleep measures*** |  |  |  |  |  |
| Sleep continuity (yes), n (%)^d^ | 2,085 (62.6) | 3,131 (59.6) | 5,216 (56.8) | 0.007 | 603 |
| Excessive daytime sleepiness (score ≥11), n (%)^e^ | 461 (13.8) | 749 (14.3) | 1,210 (13.2) | 0.549 | 619 |
| ***Cognitive functioning*** |  |  |  |  |  |
| Overall cognition, mean (SD) | 0.09 (0.63) | -0.03 (0.70) | 0.02 (0.68) | <0.001 | 565 |
| Memory, mean (SD) | 0.10 (0.95) | -0.06 (1.02) | 0.00 (1.00) | <0.001 | 402 |
| Information processing speed, mean (SD) | 0.10 (0.93) | -0.06 (1.04) | 0.00 (1.00) | <0.001 | 463 |
| Executive functioning and attention, mean (SD) | 0.08 (0.95) | -0.05 (1.03) | 0.00 (1.00) | <0.001 | 498 |
| ***Markers of brain morphology*** |  |  |  |  |  |
| Grey matter volume (ml), mean (SD) | 660.9 (59.4) | 661.3 (63.6) | 661.1 (60.9) | 0.840 | 4,004 |
| White matter volume (ml), mean (SD) | 476.0 (58.1) | 474.9 (60.4) | 475.6 (58.1) | 0.527 | 4,004 |
| Right hippocampal volume (mm^3^), mean (SD) | 3428.5 (353.4) | 3432.0 (383.2) | 3430.0 (366.7) | 0.716 | 3,205 |
| Left hippocampal volume, mean (mm^3^) | 3409.6 (351.8) | 3407.3 (375.6) | 3408.6 (362.4) | 0.813 | 3,205 |
| Total hippocampal volume, mean (mm^3^) | 6838.1 (689.0) | 6839.3 (742.1) | 6838.6 (712.7) | 0.947 | 3,205 |
| White matter hyperintensities volume (ml), mean (SD) | 0.9 (2.9) | 1.2 (4.3) | 1.02 (3.4) | 0.002 | 4,004 |
| Cerebrospinal fluid volume (ml), mean (SD) | 251.8 (46.8) | 254.6 (50.9) | 252.8 (48.3) | 0.049 | 4,004 |
| Presence of cerebral small vessel disease, *n* (%) | 1,045 (31.1) | 563 (32.3) | 1,608 () | 0.367 | 4,086 |
| Abbreviations: SD= Standard Deviation; SEP= Socio-Economic Position; T2DM= Type 2 Diabetes Mellitus; LIBRA1= original LIfestyle for BRAin Health.  ^a^ The SEP compound score was calculated using the mean of z-scores for education, occupation, and household income, categorised into tertiles: 1 = low, 2 = middle, 3 = high.  ^b^ Time in bed ranged from 4.66 – 12.66 h/night.  ^c^ Sleep breaks ranged from 0 – 13 interruptions/night.  ^d^ Based on n= 3,333 due to missing values.  ^e^ Based on n= 3,331 due to missing values.  ^*^ Participants may not have all 11 LIBRA1 factors available, leading to a potentially lower score. | | | | | |

**Table S4.** Associations between objective sleep breaks and cognitive functioning.

| **Cognitive domain** | **Sleep breaks (interruptions/night) ^a^** | | | |
| --- | --- | --- | --- | --- |
|  | **1** |  | **≥2** |  |
|  | B [95% CI] | **P-value** | B [95% CI] | **P-value** |
| ***Overall cognition*** |  |  |  |  |
| Crude model | **-0.121 [-0.166, -0.075]** | **<0.001** | **-0.325 [-0.393, -0.257]** | **<0.001** |
| Model 1 | -0.017 [-0.054, 0.019] | 0.344 | **-0.083 [-0.139, -0.029]** | **0.003** |
| Model 2 | -0.011 [-0.047, 0.026] | 0.571 | **-0.072 [-0.127, -0.016]** | **0.011** |
| Model 3 | -0.007 [-0.044, 0.029] | 0.675 | **-0.069 [-0.124, -0.013]** | **0.015** |
| ***Memory*** |  |  |  |  |
| Crude model | **-0.126 [-0.195, -0.057]** | **<0.001** | **-0.394 [-0.498, -0.290]** | **<0.001** |
| Model 1 | 0.020 [-0.041, 0.080] | 0.525 | -0.065 [-0.157, 0.027] | 0.163 |
| Model 2 | 0.025 [-0.036, 0.086] | 0.420 | -0.056 [-0.149, 0.036] | 0.233 |
| Model 3 | 0.026 [-0.034, 0.087] | 0.395 | -0.055 [-0.147, 0.038] | 0.248 |
| ***Information processing speed*** |  |  |  |  |
| Crude model | **-0.192 [-0.258, -0.125]** | **<0.001** | **-0.442 [-0.542, -0.342]** | **<0.001** |
| Model 1 | **-0.066 [-0.122, -0.009]** | **0.022** | **-0.143 [-0.229, -0.057]** | **0.001** |
| Model 2 | -0.056 [-0.113, 0.001] | 0.052 | **-0.126 [-0.213, -0.040]** | **0.004** |
| Model 3 | -0.055 [-0.113, 0.000] | 0.052 | **-0.125 [-0.212, -0.039]** | **0.005** |
| ***Executive functioning and attention*** |  |  |  |  |
| Crude model | **-0.111 [-0.180, -0.042]** | **0.002** | **-0.307 [0.410, 0.203]** | **<0.001** |
| Model 1 | -0.024 [-0.086, 0.039] | 0.455 | -0.094 [-0.189, 0.001] | 0.052 |
| Model 2 | -0.014 [-0.076, 0.049] | 0.669 | -0.077 [-0.172, 0.019] | 0.115 |
| Model 3 | -0.005 [-0.068, 0.058] | 0.874 | -0.068 [-0.164, 0.027] | 0.160 |
| Abbreviations: CI= confidence interval; B= unstandardised regression coefficient.  ^a^ Participants with no sleep breaks (0/night) served as the reference group.  Crude Model: No covariates.  Model 1: analyses adjusted for age, sex, composite socio-economic position score, type 2 diabetes mellitus.  Model 2: Model 1+ original LIfestyle for BRAin health (LIBRA1) score.  Model 3: Model 2 + sleep medication usage, time in bed. | | | | |

**Table S5.** Associations between objective sleep breaks and markers of brain morphology.

| **Brain morphology marker^a^** | **Sleep breaks ( per night)^b^** | | | |
| --- | --- | --- | --- | --- |
|  | **1** |  | **≥2** |  |
|  | B [95% CI] | **P-value** | B [95% CI] | **P-value** |
| ***Grey matter volume*** |  |  |  |  |
| Crude Model | **-0.071 [-0.107, -0.036]** | **<0.001** | **-0.215 [-0.269, -0.161]** | **<0.001** |
| Model 1 | -0.019 [-0.052, 0.014] | 0.254 | **-0.089 [-0.139, -0.039]** | **<0.001** |
| Model 2 | -0.007 [-0.040, 0.025] | 0.658 | **-0.070 [-0.119, -0.020]** | **0.009** |
| Model 3 | -0.005 [-0.037, 0.028] | 0.781 | **-0.068 [-0.118, -0.018]** | **0.007** |
| ***White matter volume*** |  |  |  |  |
| Crude Model | -0.021 [-0.056, 0.013] | 0.221 | -0.046 [-0.098, 0.005] | 0.079 |
| Model 1 | 0.003 [-0.030, 0.037] | 0.847 | 0.014 [-0.037, 0.065] | 0.588 |
| Model 2 | 0.007 [-0.027, 0.041] | 0.688 | 0.020 [-0.031, 0.072] | 0.441 |
| Model 3 | 0.007 [-0.027, 0.041] | 0.690 | 0.021 [-0.031, 0.072] | 0.428 |
| ***Right hippocampal volume*** |  |  |  |  |
| Crude Mode | **-0.065 [-0.121, -0.008]** | **0.025** | **-0.180 [-0.265, -0.095]** | **<0.001** |
| Model 1 | -0.008 [-0.062, 0.045] | 0.764 | -0.040 [-0.122, 0.041] | 0.334 |
| Model 2 | -0.004 [-0.058, 0.050] | 0.877 | -0.034 [-0.116, 0.049] | 0.423 |
| Model 3 | -0.002 [-0.056, 0.052] | 0.940 | -0.031 [-0.114, 0.051] | 0.455 |
| ***Left hippocampal volume*** |  |  |  |  |
| Crude Model | **-0.064 [-0.120, -0.007]** | **0.027** | **-0.189 [-0.274, -0.103]** | **<0.001** |
| Model 1 | -0.003 [-0.056, 0.050] | 0.909 | -0.040 [-0.121, 0.041] | 0.335 |
| Model 2 | 0.001 [-0.052, 0.054] | 0.969 | -0.030 [-0.114, 0.049] | 0.430 |
| Model 3 | 0.002 [-0.052, 0.055] | 0.948 | -0.032 [-0.113, 0.050] | 0.444 |
| ***Total hippocampal volume*** |  |  |  |  |
| Crude Model | **-0.066 [-0.122, -0.010]** | **0.021** | **-0.189 [-0.273, -0.104]** | **<0.001** |
| Model 1 | -0.006 [-0.058, 0.046] | 0.828 | -0.041 [-0.121, 0.039] | 0.314 |
| Model 2 | -0.002 [-0.054, 0.051] | 0.951 | -0.034 [-0.114, 0.046] | 0.407 |
| Model 3 | -0.000 [-0.053, 0.053] | 0.995 | -0.032 [-0.113, 0.048] | 0.430 |
| ***White matter hyperintensity volume*** *^c^* |  |  |  |  |
| Crude Model | 0.038 [-0.030, 0.106] | 0.276 | **0.213 [0.111, 0.316]** | **<0.001** |
| Model 1 | -0.026 [-0.089, 0.038] | 0.426 | 0.052 [-0.045, 0.149] | 0.291 |
| Model 2 | -0.043 [-0.107, 0.020] | 0.181 | 0.023 [-0.074, 0.119] | 0.647 |
| Model 3 | -0.041 [-0.105, 0.022] | 0.202 | 0.025 [-0.072, 0.122] | 0.616 |
| ***Cerebrospinal fluid volume*** |  |  |  |  |
| Crude Model | **0.112 [0.058, 0.165]** | **<0.001** | **0.320 [0.239, 0.401]** | **<0.001** |
| Model 1 | 0.017 [-0.028, 0.063] | 0.453 | **0.093 [0.024, 0.163]** | **0.009** |
| Model 2 | -0.001 [-0.046, 0.044] | 0.965 | 0.060 [-0.009, 0.130] | 0.079 |
| Model 3 | -0.004 [-0.050, 0.041] | 0.848 | 0.059 [-0.010, 0.129] | 0.094 |
| ***Presence of cerebral small vessel disease (yes/no)*** |  |  |  |  |
| Crude Model | OR: 1.123 [0.959, 1.315] | 0.150 | **OR: 1.266 [1.003, 1.598]** | **0.047** |
| Model 1 | OR: 0.982 [0.829, 1.164] | 0.837 | OR: 0.892 [0.694, 1.148] | 0.376 |
| Model 2 | OR: 0.954 [0.805, 1.132] | 0.590 | OR: 0.853 [0.662, 1.100] | 0.221 |
| Model 3 | OR: 0.962 [0.811, 1.142] | 0.669 | OR: 0.862 [0.668, 1.112] | 0.252 |
| Abbreviations: CI= confidence interval; B= unstandardised regression coefficient; OR = odds ratio.  ^a^ Standardised brain volume (z-scores).  ^b^ Participants with no sleep breaks (0/night) served as the reference group.  ^c^ White matter hyperintensities were transformed into a logarithmic scale.  Crude model: adjusted for magnetic resonance imaging (MRI) lag time and intracranial volume for volumetric MRI markers.  Model 1: analyses adjusted for age, sex, composite socio-economic position score, type 2 diabetes mellitus.  Model 2: Model 1+ original LIfestyle for BRAin health (LIBRA1) score.  Model 3: Model 2 + sleep medication usage, time in bed. | | | | |

**Table S6.** Associations between subjective sleep parameters and cognitive functioning.

| **Cognitive domain** | **Sleep continuity (yes)** | **P-value** | **Excessive daytime sleepiness (score ≥11)** | **P-value** |
| --- | --- | --- | --- | --- |
|  | B [95% CI] |  | B [95% CI] |  |
| ***Overall cognition*** |  |  |  |  |
| Crude model | -0.027 [-0.071, 0.173] | 0.234 | 0.027 [-0.035, 0.088] | 0.397 |
| Model 1 | -0.008 [-0.043, 0.028] | 0.675 | -0.018 [-0.066, 0.031] | 0.470 |
| Model 2 | -0.007 [-0.042, 0.028] | 0.699 | -0.013 [-0.062, 0.035] | 0.595 |
| Model 3 | -0.009 [-0.044, 0.026] | 0.620 | -0.013 [-0.062, 0.035] | 0.590 |
| ***Memory*** |  |  |  |  |
| Crude model | **-0.088 [-0.155, -0.021]** | **0.010** | 0.040 [-0.054, 0.134] | 0.400 |
| Model 1 | -0.004 [-0.063, 0.054] | 0.890 | -0.004 [-0.085, 0.077] | 0.925 |
| Model 2 | -0.003 [-0.062, 0.056] | 0.921 | -0.005 [-0.082, 0.080] | 0.990 |
| Model 3 | -0.004 [-0.063, 0.055] | 0.899 | -0.001[-0.082, 0.080] | 0.987 |
| ***Information processing speed*** |  |  |  |  |
| Crude model | -0.049 [-0.114, 0.016] | 0.137 | 0.008 [-0.083, 0.099] | 0.859 |
| Model 1 | -0.045 [-0.100, 0.009] | 0.104 | -0.054 [-0.130, 0.021] | 0.158 |
| Model 2 | -0.045 [-0.100, 0.010] | 0.108 | -0.048 [-0.123, 0.028] | 0.218 |
| Model 3 | -0.049 [-0.104, 0.006] | 0.078 | -0.047 [-0.124, 0.028] | 0.214 |
| ***Executive functioning and attention*** |  |  |  |  |
| Crude model | 0.053 [-0.013, 0.119] | 0.118 | 0.043 [-0.050, 0.136] | 0.365 |
| Model 1 | 0.021 [-0.039, 0.082] | 0.489 | -0.008 [-0.092, 0.075] | 0.842 |
| Model 2 | 0.021 [-0.039, 0.082] | 0.485 | -0.002 [-0.085, 0.082] | 0.967 |
| Model 3 | 0.019 [-0.041, 0.080] | 0.528 | -0.002 [-0.085, 0.081] | 0.964 |
| Abbreviations: CI= confidence interval; B= unstandardised regression coefficient.  Crude Model: No covariates.  Model 1: analyses adjusted for age, sex, socio-economic position, type 2 diabetes mellitus.  Model 2: Model 1+ original LIfestyle for BRAin health (LIBRA1) score.  Model 3: Model 2 + sleep medication usage. | | | | |

**Table S7.** Associations between subjective sleep parameters and markers of brain morphology.

| **Brain morphology marker**^a^ | **Sleep continuity (yes)** | **P-value** | **Excessive daytime sleepiness (score ≥ 11)** | **P-value** |
| --- | --- | --- | --- | --- |
|  | B [95% CI] |  | B [95% CI] |  |
| ***Grey matter volume*** |  |  |  |  |
| Crude Model | -0.003 [-0.038, 0.032] | 0.864 | 0.018 [-0.031, 0.067] | 0.469 |
| Model 1 | -0.009 [-0.040, 0.023] | 0.587 | -0.015 [-0.058, 0.029] | 0.526 |
| Model 2 | -0.008 [-0.040, 0.023] | 0.603 | -0.005 [-0.049, 0.038] | 0.812 |
| Model 3 | -0.006 [-0.038, 0.025] | 0.700 | -0.005 [-0.049, 0.038] | 0.819 |
| ***White matter volume*** |  |  |  |  |
| Crude Model | 0.002 [-0.031, 0.036] | 0.896 | 0.003 [-0.043, 0.050] | 0.885 |
| Model 1 | -0.004 [-0.037, 0.028] | 0.790 | -0.014 [-0.059, 0.031] | 0.547 |
| Model 2 | -0.003 [-0.036, 0.029] | 0.846 | -0.011 [-0.056, 0.034] | 0.621 |
| Model 3 | -0.005 [-0.038, 0.028] | 0.770 | -0.011 [-0.056, 0.034] | 0.616 |
| ***Right hippocampal volume*** |  |  |  |  |
| Crude Model | 0.004 [-0.052, 0.059] | 0.898 | 0.055 [-0.021, 0.132] | 0.158 |
| Model 1 | -0.015 [-0.067, 0.037] | 0.580 | 0.014 [-0.058, 0.085] | 0.708 |
| Model 2 | -0.013 [-0.065, 0.039] | 0.616 | 0.017 [-0.055, 0.089] | 0.641 |
| Model 3 | -0.014 [-0.066, 0.038] | 0.591 | 0.017 [-0.055, 0.089] | 0.643 |
| ***Left hippocampal volume*** |  |  |  |  |
| Crude Model | -0.012 [-0.067, 0.044] | 0.678 | 0.019 [-0.057, 0.096] | 0.621 |
| Model 1 | -0.029 [-0.081, 0.022] | 0.264 | -0.025 [-0.095, 0.046] | 0.498 |
| Model 2 | -0.028 [-0.080, 0.023] | 0.280 | -0.021 [-0.092, 0.050] | 0.560 |
| Model 3 | -0.029 [-0.081, 0.022] | 0.266 | -0.021 [-0.092, 0.050] | 0.558 |
| ***Total hippocampal volume*** |  |  |  |  |
| Crude Model | -0.004 [-0.059, 0.050] | 0.883 | 0.038 [-0.037, 0.114] | 0.321 |
| Model 1 | -0.022 [-0.073, 0.028] | 0.385 | -0.005 [-0.075, 0.064] | 0.879 |
| Model 2 | -0.021 [-0.072, 0.029] | 0.411 | -0.004 [-0.074, 0.066] | 0.913 |
| Model 3 | -0.022 [-0.073, 0.029] | 0.391 | -0.002 [-0.072, 0.068] | 0.954 |
| ***White matter hyperintensity volume*** *^b^* |  |  |  |  |
| Crude Model | -0.031 [-0.098, 0.036] | 0.363 | -0.001 [-0.094, 0.091] | 0.980 |
| Model 1 | 0.011 [-0.050, 0.072] | 0.728 | 0.055 [-0.030, 0.139] | 0.206 |
| Model 2 | 0.009 [-0.052, 0.070] | 0.776 | 0.042 [-0.042, 0.127] | 0.329 |
| Model 3 | 0.007 [-0.054, 0.069] | 0.817 | 0.042 [-0.043, 0.126] | 0.331 |
| ***Cerebrospinal fluid volume*** |  |  |  |  |
| Crude Model | 0.003 [-0.050, 0.056] | 0.915 | -0.025 [-0.098, 0.048] | 0.505 |
| Model 1 | 0.016 [-0.028, 0.061] | 0.464 | 0.035 [-0.026, 0.095] | 0.264 |
| Model 2 | 0.015 [-0.029, 0.059] | 0.515 | 0.021 [-0.040, 0.082] | 0.500 |
| Model 3 | 0.014 [-0.030, 0.058] | 0.533 | 0.021 [-0.040, 0.082] | 0.501 |
| ***Presence of cerebral small vessel disease (yes/no)*** |  |  |  |  |
| Crude Model | OR: 0.877 [0.754, 1.020] | 0.090 | OR: 1.047 [0.847, 1.293] | 0.671 |
| Model 1 | OR: 0.915 [0.777, 1.078] | 0.289 | OR: 1.205 [0.962, 1.511] | 0.105 |
| Model 2 | OR: 0.914 [0.776, 1.076] | 0.279 | OR: 1.184 [0.943, 1.485] | 0.145 |
| Model 3 | OR: 0.908 [0.770, 1.070] | 0.248 | OR: 1.184 [0.943, 1.486] | 0.145 |
| Abbreviations: CI= confidence interval; B= unstandardised regression coefficient; OR= odds ratio.  ^a^ Standardised brain volume (z-scores).  ^b^ White matter hyperintensities were transformed into a logarithmic scale.  Crude model: adjusted for magnetic resonance imaging (MRI) lag time and intracranial volume for volumetric MRI markers.  Model 1: analyses adjusted for age, sex, composite socio-economic position score, type 2 diabetes mellitus.  Model 2: Model 1+ original LIfestyle for BRAin health (LIBRA1) score.  Model 3: Model 2 + sleep medication usage. | | | | |

**Table S8**. Associations between objective time in bed and cognitive functioning, regardless of whether they have magnetic resonance imaging data available or not.

|  | **Time in bed (h/night) (continuous)** | | **Time in bed (categories^a^)** | | | |
| --- | --- | --- | --- | --- | --- | --- |
|  |  | **P-value** | **Short (<7 h/night)** | **P-value** | **Long** **(≥9 h/night)** | **P-value** |
|  | B [95% CI] |  | B [95% CI] |  | B [95% CI] |  |
| ***Overall cognition*** |  |  |  |  |  |  |
| Model 3 | **-0.018 [-0.033, -0.003]** | **0.022** | -0.005 [-0.055, 0.044] | 0.833 | **-0.041 [-0.079, -0.004]** | **0.032** |
| ***Memory*** |  |  |  |  |  |  |
| Model 3 | -0.024 [-0.049, 0.002] | 0.066 | 0.027 [-0.054, 0.108] | 0.517 | -0.035 [-0.097, 0.027] | 0.265 |
| ***Information processing speed*** |  |  |  |  |  |  |
| Model 3 | -0.002 [-0.026, 0.022] | 0.860 | -0.070 [-0.146, 0.007] | 0.074 | -0.056 [-0.115, 0.002] | 0.059 |
| Model 3 (quadratic term)^b^ | **-0.020 [-0.036, -0.003]** | **0.018** | - | - | - | - |
| ***Executive functioning and attention*** |  |  |  |  |  |  |
| Model 3 | **-0.036 [-0.062, -0.010]** | **0.006** | 0.017 [-0.067, 0.100] | 0.696 | -0.056 [-0.119, 0.008] | 0.086 |
| Abbreviations: CI= confidence interval, B= unstandardised regression coefficient.  ^a^ A mid-range (≥7 to <9 h/night) time in bed served as a reference group.  ^b^ A quadratic term for time in bed was additionally included, demonstrating a better fit than the simpler Model 3, which used only the linear term.  Model 3: analyses adjusted for age, sex, composite socio-economic position score, type 2 diabetes mellitus, original LIfestyle for BRAin health (LIBRA1) score, sleep medication usage. | | | | | | |

**Table S9.** Associations between objective sleep breaks and cognitive functioning, regardless of whether they have magnetic resonance imaging data available or not.

| **Cognitive domain** | **Sleep breaks (per night) ^a^** | | | |
| --- | --- | --- | --- | --- |
|  | **1** |  | **≥2** |  |
|  | B [95% CI] | **P-value** | B [95% CI] | **P-value** |
| ***Overall cognition*** |  |  |  |  |
| Model 3 | -0.014 [-0.043, 0.017] | 0.376 | **-0.058 [-0.103, -0.014]** | **0.010** |
| ***Memory*** |  |  |  |  |
| Model 3 | 0.018 [-0.032, 0.069] | 0.476 | -0.029 [-0.103, 0.045] | 0.443 |
| ***Information processing speed*** |  |  |  |  |
| **Model 3** | **-0.051 [-0.098, -0.003]** | **0.036** | **-0.104 [-0.173, -0.034]** | **0.003** |
| ***Executive functioning and attention*** |  |  |  |  |
| Model 3 | -0.023 [-0.075, 0.029] | 0.380 | **-0.081 [-0.157, -0.005]** | **0.036** |
| Abbreviations: CI= confidence interval; B= unstandardised regression coefficient.  ^a^ Participants with no sleep breaks (0/night) served as the reference group.  Model 3: analyses adjusted for age, sex, composite socio-economic position score, type 2 diabetes mellitus, original LIfestyle for BRAin health (LIBRA1) score, time in bed, sleep medication usage. | | | | |

**Table S10.** Associations between subjective sleep parameters and cognitive functioning, regardless of whether they have magnetic resonance imaging data available or not.

| **Cognitive domain** | **Sleep continuity (yes)** | **P-value** | **Excessive daytime sleepiness (score ≥11)** | **P-value** |
| --- | --- | --- | --- | --- |
|  | B [95% CI] |  | B [95% CI] |  |
| ***Overall cognition*** |  |  |  |  |
| Model 3 | 0.003 [-0.027, 0.032] | 0.862 | 0.002 [-0.039, 0.043] | 0.925 |
| ***Memory*** |  |  |  |  |
| Model 3 | 0.008 [-0.040, 0.056] | 0.754 | 0.002 [-0.065, 0.069] | 0.960 |
| ***Information processing speed*** |  |  |  |  |
| Model 3 | -0.028 [-0.073, 0.017] | 0.226 | -0.014 [-0.077, 0.050] | 0.670 |
| ***Executive functioning and attention*** |  |  |  |  |
| Model 3 | 0.028 [-0.022, 0.077] | 0.271 | 0.018 [-0.050, 0.087] | 0.598 |
| Abbreviations: CI= confidence interval; B= unstandardised regression coefficient.  Model 3: analyses adjusted for age, sex, composite socio-economic position score, type 2 diabetes mellitus, original LIfestyle for BRAin health (LIBRA1) score, sleep medication usage. | | | | |

**SENSITIVITY ANALYSES (Text S1)**

Another sensitivity analysis was conducted in which the socio-economic position (SEP) components—educational attainment, household income, and occupational status —were included separately in the main model (Model 3). Overall, findings remained largely consistent; however, some differences emerged. Specifically, a longer time in bed (TIB) was no longer associated with worse executive functioning and attention (B= -0.038, 95%CI= -0.132 to 0.055, p=0.425). A linear association between TIB and grey matter volume remained (B_linear_= -0.028, 95%CI= -0.048 to -0.008, p=0.007), but a curvilinear association was no longer statistically significant (B_quadratic_= -0.014, 95%CI= -0.028 to 0.001, p=0.063).

In addition, new findings surfaced. A curvilinear association was observed between TIB and right hippocampal volume (B_quadratic_= -0.039, 95%CI= -0.048 to 0.008, p=0.007). Categorical analyses further showed that those with a long TIB had lower right hippocampal volume (B= -0.087, 95%CI= -0.167 to -0.007, p=0.033). Additionally, participants with a long TIB had significantly lower odds of cerebral small vessel disease (OR= 0.749, 95%CI= 0.574 to 0.976, p=0.033). While the main analyses revealed no significant associations for subjective sleep parameters, the sensitivity analyses showed that sleep continuity was negatively associated with information processing speed (B= -0.064, 95%CI= -0.128 to -0.001, p=0.047) and lower left hippocampal volume (B= -0.062, 95%CI= -0.123 to -0.002, p=0.043). Furthermore, excessive daytime sleepiness was associated with worse information processing (B= -0.093, 95%CI= -0.178 to -0.008, p=0.032).

Differences in findings may reflect reduced power in the sensitivity analyses (n= 2,459 vs. n= 3,360) and potential selection bias due to greater missingness when SEP components, particularly household income, were modelled separately. Income is frequently underreported due to privacy concerns and its sensitive nature, leading to non-random exclusion. While disaggregating SEP may offer conceptual clarity, it can also increase measurement variability, particularly given that SEP is a multidimensional construct with overlapping components.

**REFERENCES**

1. Heger IS, Deckers K, Schram MT, et al. Associations of the Lifestyle for Brain Health Index With Structural Brain Changes and Cognition: Results From the Maastricht Study. *Neurology*. 2021; 97 (13): e1300-e1312. doi:[10.1212/WNL.0000000000012572](https://doi.org/10.1212/wnl.0000000000012572)

2. van Dongen MCJM, Wijckmans-Duysens NEG, den Biggelaar LJCJ, et al. The Maastricht FFQ: Development and validation of a comprehensive food frequency questionnaire for the Maastricht study. *Nutrition*. 2019; 62: 39-46. doi: [10.1016/j.nut.2018.10.015](https://doi.org/10.1016/j.nut.2018.10.015)

3. Trichopoulou A, Orfanos P, Norat T, et al. Modified Mediterranean diet and survival: EPIC-elderly prospective cohort study. *BMJ*. 2005; 330 (7498): 991. doi:[10.1136/bmj.38415.644155.8F](https://doi.org/10.1136/bmj.38415.644155.8f)

4. Resnicow K, McCarty F, Blissett D, Wang T, Heitzler C, Lee RE. Validity of a modified CHAMPS physical activity questionnaire among African-Americans. *Med Sci Sports Exerc*. 2003; 35 (9): 1537-1545. doi:[10.1136/bmj.38415.644155.8F](https://doi.org/10.1136/bmj.38415.644155.8f)

5. Weggemans RM, Backx FJ, Borghouts L, et al. The 2017 Dutch physical activity guidelines. *Int J Behav Nutr Phys Act*. 2018; 15: 1-12. doi:[10.1186/s12966-018-0661-9](https://doi.org/10.1186/s12966-018-0661-9)

6. Kromhout D, Spaaij C, de Goede J, Weggemans R. The 2015 Dutch food-based dietary guidelines. *Eur J Clin Nutr.* 2016; 70 (8): 869-878. doi:[10.1038/ejcn.2016.52](https://doi.org/10.1038/ejcn.2016.52)

7. World Health Organization. Obesity and overweight. https://www.who.int/news-room/fact-sheets/detail/obesity-and-overweight. Accessed 23-01, 2025.

8. Sheehan DV, Lecrubier Y, Sheehan KH, et al. The Mini-International Neuropsychiatric Interview (M.I.N.I.): the development and validation of a structured diagnostic psychiatric interview for DSM-IV and ICD-10. *J Clin Psychiatry.* 1998; 59 Suppl 20: 22-33.

9. Kroenke K, Spitzer RL, Williams JB. The PHQ‐9: validity of a brief depression severity measure. J Gen Intern Med. 2001; 16 (9): 606-613. doi: [10.1046/j.1525-1497.2001.016009606.x](https://doi.org/10.1046/j.1525-1497.2001.016009606.x)

10. World Health Organization. Definition and diagnosis of diabetes mellitus and intermediate hyperglycaemia : report of a WHO/IDF consultation. In. Geneva: World Health Organization; 2006.

11. Rose G, McCartney P, Reid D. Self-administration of a questionnaire on chest pain and intermittent claudication. *Br J Prev Soc Med.* 1977; 31 (1): 42-48. doi: [10.1136/jech.31.1.42](https://doi.org/10.1136/jech.31.1.42)

12. Inker LA, Schmid CH, Tighiouart H, et al. Estimating glomerular filtration rate from serum creatinine and cystatin C. *N Engl J Med.* 2012; 367 (1): 20-29. doi: [10.1056/NEJMoa1114248](https://doi.org/10.1056/nejmoa1114248)
